# Hybrid de novo genome assembly of red gromwell (*Lithospermum erythrorhizon*) reveals evolutionary insight into shikonin biosynthesis

**DOI:** 10.1038/s41438-020-0301-9

**Published:** 2020-06-01

**Authors:** Robert P. Auber, Thiti Suttiyut, Rachel M. McCoy, Manoj Ghaste, Joseph W. Crook, Amanda L. Pendleton, Joshua R. Widhalm, Jennifer H. Wisecaver

**Affiliations:** 1 Department of Biochemistry, Purdue University, West Lafayette, IN 47907 USA; 2 Purdue Center for Plant Biology, Purdue University, West Lafayette, IN 47907 USA; 3 Department of Horticulture and Landscape Architecture, Purdue University, West Lafayette, IN 47907 USA

**Keywords:** Secondary metabolism, Phylogenomics

## Abstract

*Lithospermum erythrorhizon* (red gromwell; zicao) is a medicinal and economically valuable plant belonging to the Boraginaceae family. Roots from *L. erythrorhizon* have been used for centuries based on the antiviral and wound-healing properties produced from the bioactive compound shikonin and its derivatives. More recently, shikonin, its enantiomer alkannin, and several other shikonin/alkannin derivatives have collectively emerged as valuable natural colorants and as novel drug scaffolds. Despite several transcriptomes and proteomes having been generated from *L. erythrorhizon*, a reference genome is still unavailable. This has limited investigations into elucidating the shikonin/alkannin pathway and understanding its evolutionary and ecological significance. In this study, we obtained a de novo genome assembly for *L. erythrorhizon* using a combination of Oxford Nanopore long-read and Illumina short-read sequencing technologies. The resulting genome is ∼367.41 Mb long, with a contig N50 size of 314.31 kb and 27,720 predicted protein-coding genes. Using the *L. erythrorhizon* genome, we identified several additional *p*-hydroxybenzoate:geranyltransferase (PGT) homologs and provide insight into their evolutionary history. Phylogenetic analysis of prenyltransferases suggests that PGTs originated in a common ancestor of modern shikonin/alkannin-producing Boraginaceous species, likely from a retrotransposition-derived duplication event of an ancestral prenyltransferase gene. Furthermore, knocking down expression of *LePGT1* in *L. erythrorhizon* hairy root lines revealed that LePGT1 is predominantly responsible for shikonin production early in culture establishment. Taken together, the reference genome reported in this study and the provided analysis on the evolutionary origin of shikonin/alkannin biosynthesis will guide elucidation of the remainder of the pathway.

## Introduction

**Fig. 1:**
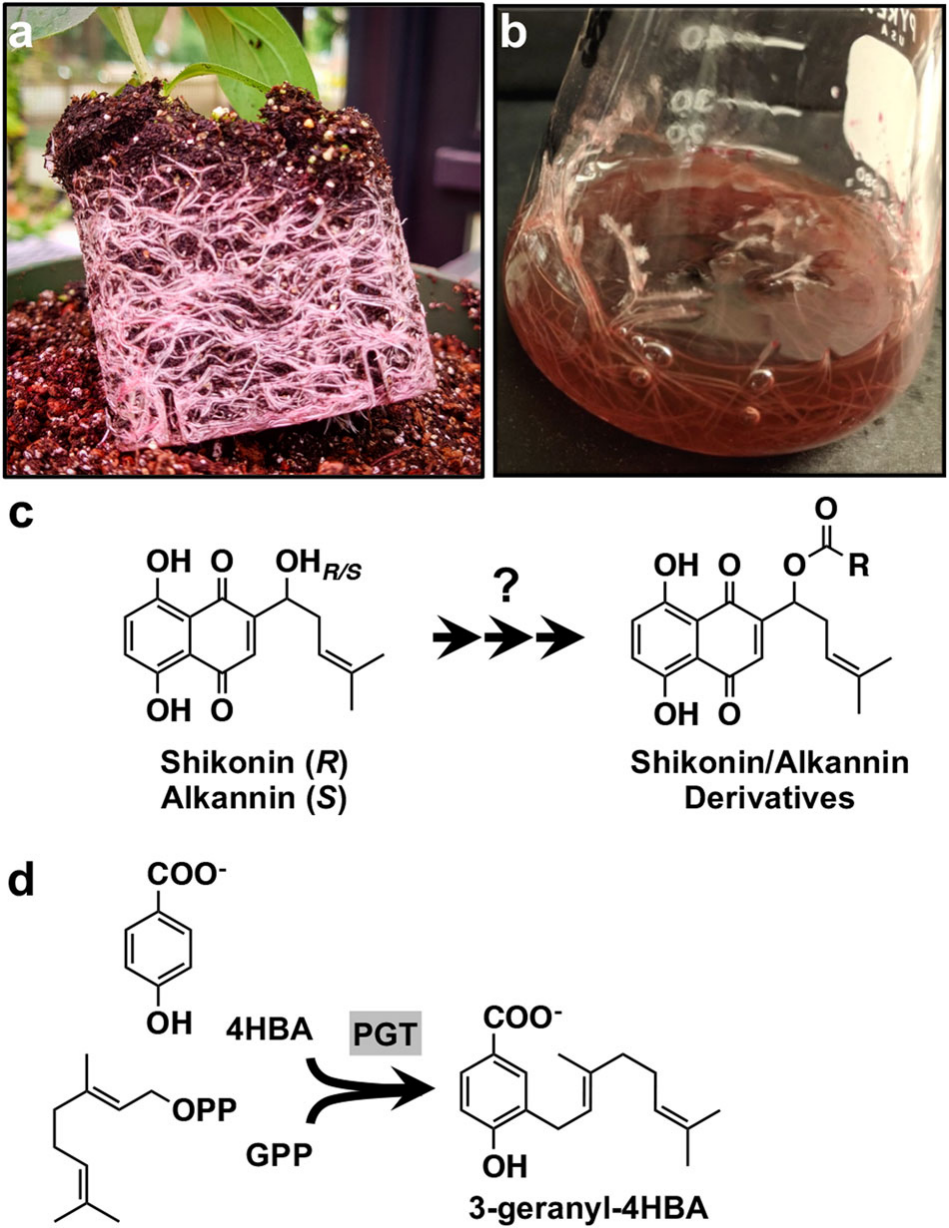
Shikonin is produced in the roots of *Lithospermum erythrorhizon*. **a** Intact roots of *L. erythrorhizon* producing shikonin. **b** Hairy root culture of *L. erythrorhizon* producing shikonin. **c** The structures of shikonin and its enantiomer, alkannin. Shikonin and alkannin are precursor to dozens of acylated derivatives collectively produced by members of the Boraginaceae. **d** The shikonin pathway starts with the conjugation of 4-hydroxybenzoic acid (4HBA) and geranyl diphosphate (GPP) catalyzed by *p*-hydroxybenzoate:geranyltransferase (PGT)

The purple-colored roots of red gromwell (*Lithospermum erythrorhizon*; Fig. 1a, b), also known as “zicao” in Chinese, “jichi” in Korean, and “murasaki” in Japanese, have been used as part of traditional medicines, as a dyestuff, and in cosmetics across many cultures for centuries. The responsible bioactive and pigmented compounds, shikonin—or its enantiomer, alkannin—and dozens of other acylated shikonin/alkannin derivatives (Fig. 1c), are synthesized in the root periderm of *L. erythrorhizon* and several other Boraginaceae species^1,2^. Shikonins/alkannins are deposited into the rhizosphere where they function in plant-microbe interactions and interfere with the growth of competing plants (allelopathy), roles suggested to have contributed to the invasion success of species like *Echium plantagineum*^3^. The presence of alkannins was also reported in *Plagiobothrys arizonicus* leaves^4^, though the physiological and/or ecological significance of their presence in aerial tissues is unclear.

Shikonins/alkannins have more recently been discovered to exhibit a range of pharmacological properties^5^. Shikonin has been found to suppress human immunodeficiency virus (HIV) type 1^6^ and to display anti-tumor effects in breast cancer cells via multiple signaling pathways^7^. Thus, combined with their traditional medicinal and cosmetic value, there has been wide interest for many decades in scaling shikonin/alkannin production. Early efforts back in the 1970s and 1980s centered on producing shikonin in *L. erythrorhizon* cell cultures, which was also the first industrial scale platform for producing a secondary metabolite in dedifferentiated plant cells^8^. With advances in understanding of pathway precursors^4,9^, strategies to increase shikonin production in *L. erythrorhizon* through metabolic engineering were developed (e.g.,^10,11^). Current efforts have extended to include synthetic chemistry for producing shikonin, alkannin, and derivatives with higher specificity and potency^12,13^. Moreover, the use of comparative transcriptomics (e.g.,^14–16^) and proteomics^17^ approaches for elucidating the shikonin/alkannin pathway has uncovered several gene candidates and led to the identification of the geranylhydroquinone hydroxylase (GHQH; CYP76B74)^18^. Comparatively less attention has focused on the evolutionary origin of shikonin/alkannin pathway genes^16,19^ and on the ecological significance of producing the compounds^3^. It was only recently that the *E. plantagineum* genome was published, the first from a shikonin/alkannin-producing species or a member of the Boraginales^20^.

Despite being among the longest-studied plant natural products, there are still many gaps in knowledge about shikonin/alkannin pathway genes, architecture, and regulation. In this study, we report the first de novo genome for *L. erythrorhizon*, ﻿generated by combining Oxford Nanopore Technology (ONT) long reads with Illumina HiSeq short reads. From this assembly, we identified the existence of 11 previously unreported *p*-hydroxybenzoate:geranyltransferase (PGT; Fig. 1c) homologs and provide insight into their contribution to shikonin biosynthesis based on (i) their distribution amongst shikonin/alkannin-producing species within the Boraginaceae and (ii) *L. erythrorhizon* knock-down hairy root lines with reduced expression of *LePGT1*. Taken together, the results of our study provide evolutionary insight into the origin of the shikonin/alkannin pathway, and the genome assembly offers a major resource for exploring outstanding questions in shikonin/alkannin metabolism.

## Results

### Genome assembly and annotation

To create a reference genome, we combined *L. erythrorhizon* ONT genomic DNA (gDNA) reads generated in-house from Siebold & Zucc. plants with publicly available Illumina gDNA reads sequenced by Nanjing University in 2018 from an unknown accession (SRR5644206). The Illumina data consisted of ~21.7 Gb Illumina HiSeq paired-end short reads (150 bp) with an estimated heterozygosity of 0.39% and projected genome size of 369.34 Mb (Fig. S1a). Our in-house ONT data consisted of ~7.6 Gb long-reads (N50 = 15.03 kb) providing roughly 20-fold genome coverage. The short and long reads assembled into 2465 contigs using the DBG2OLC hybrid assembler^21^, yielding a 367.41 Mb genome with a longest contig of 3.44 Mb and an N50 contig length of 314.31 kb (Table 1).

**Table 1:** Summary of *L. erythrorhizon* genome assembly and gene models

*Genome assembly statistics*	
Total length	367,405,101
No. contigs	2465
Largest contig length	3,439,996
N50 contig length	314,306
N90 contig length	61,630
Counts of N50 (no. contigs)	233
Counts of N90 (no. contigs)	1370
Genome GC content	35.17%
*Gene model statistics*	
Gene number	27,720
Gene density (kb/gene)	13.25
Mean gene length	3772
Avg. no. exons per gene	7
Mean exon length	320
Exon GC content	39.32%

Using a de novo repeat modeler, 51.78% of assembly bases were denoted as repetitive elements and were subsequently masked (Table S1). Of these elements, the majority were long terminal repeats (LTRs) which comprised 23.43% of the genome assembly. Unclassified elements were the second most common, accounting for 21% of the genome. DNA repeat elements comprised 4.45% of the genome. The repeat content in *L. erythrorhizon* is comparable to the repeat content observed in the other sequenced Boraginaceae, *E. plantagineum* (43.3% repetitive; 23.08% LTRs)^20^.

Protein-coding genes were identified through a combination of ab initio, homology-based, and transcriptome-based prediction methods. A total of 27,720 genes encoding 39,395 proteins were predicted (Table 1). The average protein-coding gene was 3772 bp long and contained 7 exons. Functional annotations were assigned to 80.02%, 72.89%, 59.30%, 23.57%, 7.53%, 5.85% of genes using the InterPro^22^, Pfam^23^, GO^24^, Trans Membrane (TMHMM), KEGG, and MetaCyc^25^ databases, respectively (Supplementary Dataset S1).

### Quality assessment

To evaluate the completeness and coverage of the assembly, we aligned the ONT gDNA, Illumina gDNA, and Illumina RNA-seq reads to the *L. erythrorhizon* genome assembly. Coverage histograms of the ONT and Illumina gDNA reads showed a single peak at ~18-fold and ~45-fold coverage, respectively (Fig. S1b, c), indicative of the genome being largely homozygous. The alignment rates of the Illumina gDNA reads was high (95.97%); however 46% of the assembled genome lacked gDNA read support (Fig. S1c). This is likely due to the Illumina gDNA reads being generated via a PCR-amplified library, leading to inconsistent coverage across the genome. However, the amount of coding-regions with Illumina gDNA read support was 89.6% (≥10 mapped reads), allowing for sufficient base corrections of the ONT reads in the genic regions, which are the focus of our study. The alignment rate of the RNA reads ranged from 59.29% to 72.71% (in the case of libraries prepared via ribosomal depletion) to 86.74% to 90.15% (for libraries prepared via polyA capture) (Table S2).

We then used BUSCO^26^ to assess the completeness of the predicted proteome. Within the *L. erythrorhizon* protein-coding gene set, 1142 of 1400 conserved embryophyta genes (79.3%) were identified as complete, of those 93.43% were present in single-copy and 6.57% were duplicated (Table S3). Furthermore, 279 of 303 conserved eukaryota genes (92.08%) were identified as complete, of those 78.14% were present in single-copy and 21.86% were duplicated (Table S3). Lastly, we calculated the Alien Index (AI) for all predicted proteins of the genome assembly to assess possible contamination^27^. No assembly contig had a majority of their predicted proteins with AI scores >0, indicating no detectible contamination in the assembly. Only 50 of the 39,395 total proteins (0.13%) had AI scores >0.05, which could be indicative of horizontal gene transfer (HGT) (Supplementary Dataset S2). However, manual inspection of each of these proteins did not yield any strong HGT candidates.

### Gene family analysis

**Fig. 2:**
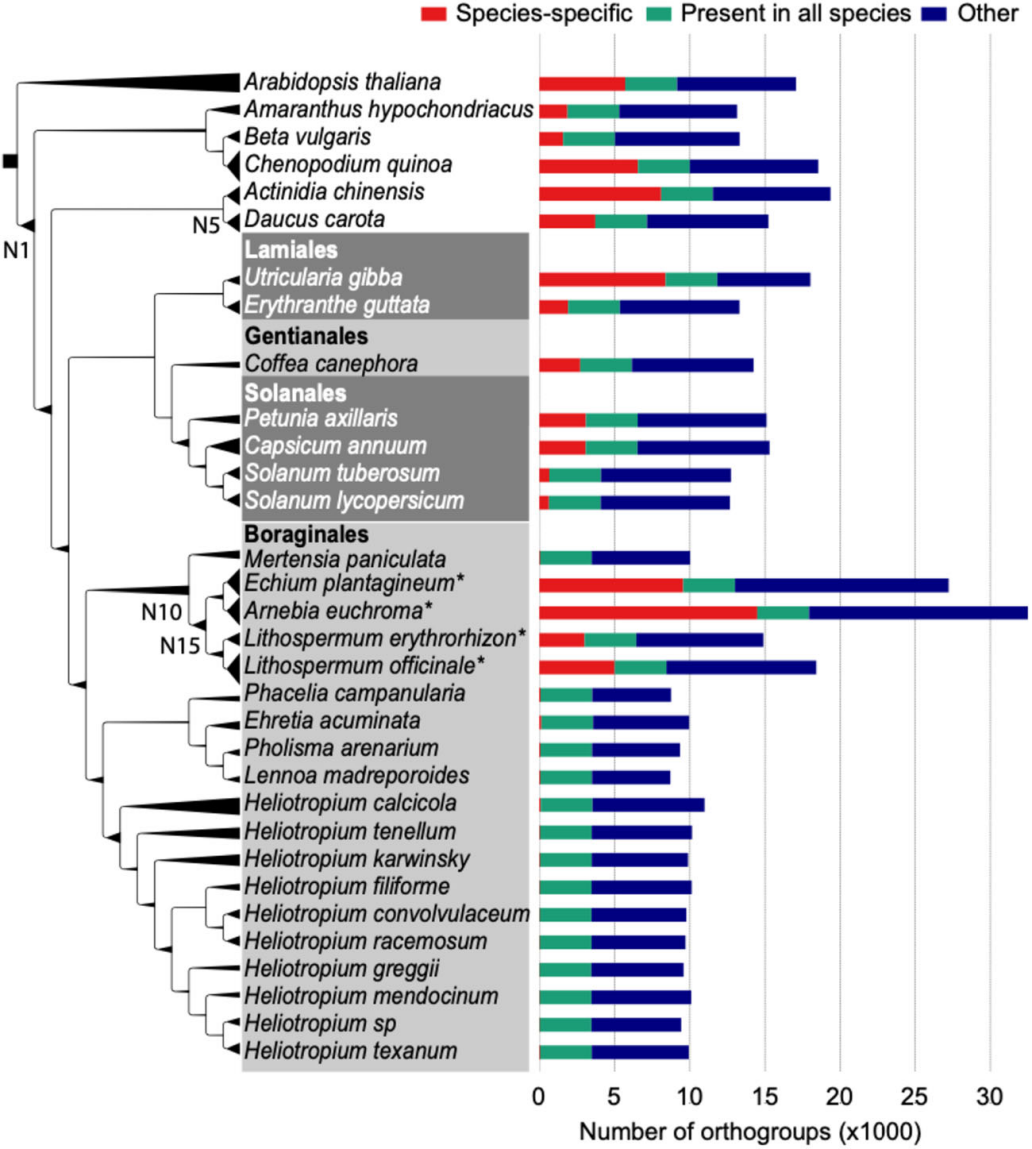
OrthoFinder gene family analysis. The OrthoFinder inferred species phylogeny is displayed on the left. The branch thickness is scaled based on the number of predicted duplicated events to have occurred at the descendent node; thinner branches indicate fewer duplications, thicker branches indicate more (see Table S7). Internodes discussed in the text (N1, N5, N10, and N15) are labeled (the species tree with all labeled internodes can be accessed in Table S7). Asterisks (*) indicate known shikonin/alkannin-producing species. Horizontal bar plots (right) indicate the number of orthogroups that are species-specific (red), maintained in all 32 species (green), or present in more than one but less than all species in the analysis (blue)

To investigate the evolution of different gene families, including those involved in the production of shikonin, we performed an OrthoFinder^28^ analysis using the protein-coding genes of *L. erythrorhizon* and 31 other eudicot species (Table S4). Incorporated into our analysis were four additional Boraginaceae species, including three species known to produce shikonin (*E. plantagineum, Arnebia euchroma*, and *Lithospermum officinale*) as well as *Mertensia paniculate*, a Boraginaceae whose transcriptome was sequenced by the 1000 Plants Initiative (oneKP^29^) and whose ability to produce shikonin is unknown. We also included 14 additional Boraginales species sequenced by the oneKP project (Table S4). Our OrthoFinder-inferred species tree had the Boraginales sister to a large clade consisting of the Solanales, Gentianales, and Laminales (Fig. 2). This placement is in disagreement with the analysis by Tang et al^20^. that showed the Boraginales sister to the Solanales. This conflict is unsurprising as the evolutionary relationships of these lamiid orders remains uncertain^30^. Additional work is needed to resolve these relationships and determine the source of the phylogenetic discordance.

The OrthoFinder analysis identified 100,874 orthogroups (predicted gene families), of which 24,346 consisted of two or more species in the analysis (Table S5). Of the 14,885 orthogroups containing one or more *L. erythrorhizon* sequences, 3441 orthogroups (23.13%) were present in all species, 3003 (20.17%) were *L. erythrorhizon* specific, and 8441 (56.71%) consisted of *L. erythrorhizon* and one or more additional species (Fig. 2; Table S6). In total, 36,392 of 39,395 *L. erythrorhizon* proteins (92.38%) were assigned to an orthogroup containing sequence(s) from one or more additional species (Table S6; Supplementary Dataset S3). The total number of *L. erythrorhizon* orthogroups (14,885) was comparable to the other sequenced genomes in the analysis (average 15,227), which ranged from 12,666 in *Solanum lycopersicum* (tomato) to 19,349 in *Actinidia chinensis* (kiwi). Furthermore, the percentage of *L. erythrorhizon* orthogroups that was species-specific (20.17%) was also comparable to the other sequenced genomes (average 22.47%), ranging from 5.05% in tomato to 46.55% in *Utricularia gibba* (a carnivorous aquatic bladderwort). The other three species known to produce shikonin showed an increased number of orthogroups (average 26,040.33), which is likely a result of their predicted proteomes being derived from de novo transcriptome assemblies rather than sequenced genomes. The average percentage of species-specific orthogroups found in the oneKP transcriptomes (0.50%) was noticeably lower that the other species in the analysis (Fig. 2) due to these proteomes being filtered prior to publication^29^.

To identify orthogroups that had expanded in one or more ancestors of *L. erythrorhizon*, we parsed the number of OrthoFinder-predicted gene duplications at each node of the inferred species tree (Fig. 2; Table S7). The average number of orthogroups that duplicated one or more times at a given internode (i.e., non-leaf node) was 1,489.55 and ranged from 41 duplications at internode N5 (the common ancestor of *Daucus carota* and *A. chinensis)* to 4489 duplications at internode N1 (the common ancestor of Caryophyllales and asterids) (Table S7). A total of 2818 orthogroups duplicated at internode N10 (the common ancestor of the five Boraginaceae species), and the *L. erythrorhizon* genes that duplicated at this internode were enriched in 21 gene ontology (GO) categories (Benjamini–Hochberg adjusted *p-*value < 0.1; Table S8) including transferase activity (GO:0016740; *p* = 1.10e−3), signal transduction (GO:0007165; *p* = 6.45e−4), and transmembrane transporter activity (GO:0022857; *p* = 0.08). Similarly, the *L. erythrorhizon* genes from the 3908 orthogroups that duplicated at internode N15 (the common ancestor of the four Boraginaceae species known to produce shikonin) were enriched in 14 GO categories (Benjamini–Hochberg adjusted *p-*value < 0.1; Table S8) including transferase activity of acyl groups (GO:0016746; *p* = 0.3), DNA-binding transcription factor activity (GO:0003700; *p* = 3.79e−3), and transmembrane transport (GO:0055085; *p* = 0.07).

### Evolution of p-hydroxybenzoate:geranyltransferase (PGT) genes for shikonin biosynthesis

One orthogroup predicted to have undergone multiple duplication events in the last common ancestor of shikonin/alkannin-producing species was OG0000509. This orthogroup was comprised of genes that code for prenyltransferases, including the characterized ubiquinone prenyltransferase in *Arabidopsis thaliana*, AtPPT1 (Coq2; At4g23660)^31^. In addition, OG0000509 contained 13 *L. erythrorhizon* genes (Table S9), including Leryth_015068 (hereafter referred to as *LePGT1*), which encodes a protein 97.06% identical to LePGT1, and Leryth_002561 (hereafter referred to as *LePGT2*), which encodes a protein 100% identical to LePGT2^32^. The *p*-hydroxybenzoate:geranyltransferases (PGTs) catalyze the conjugation of 4-hydroxybenzoic acid (4HBA) and geranyl diphosphate (GPP), the first and committed step of the shikonin pathway (Fig. 1d)^32–34^ and have been identified from several shikonin/alkannin-producing species^19,35–37^.

**Fig. 3:**
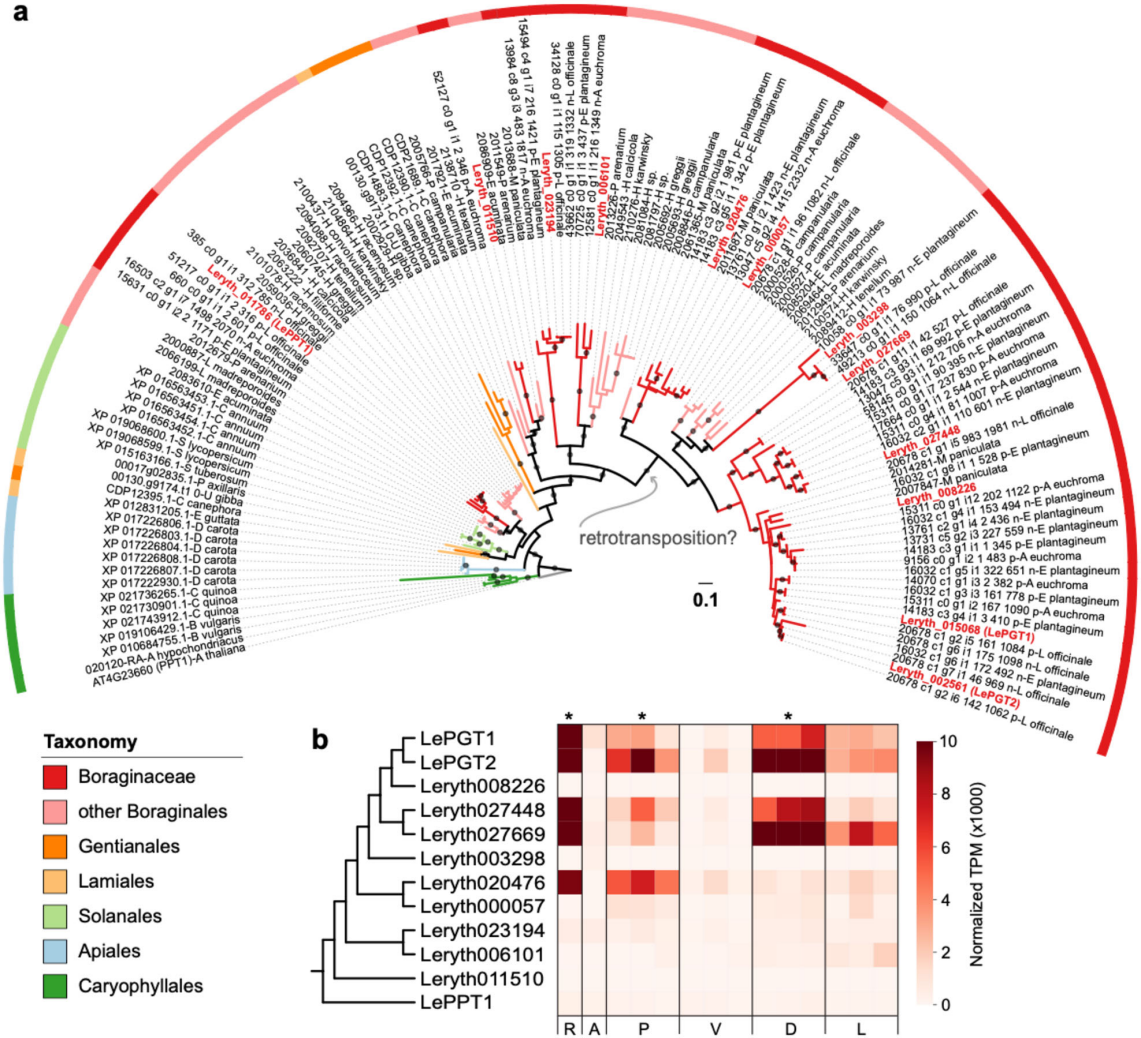
Phylogenetic analysis of prenyltransferase homologs in orthogroup OG0000509. **a** Maximum likelihood tree of orthogroup OG0000509 rooted on *Arabidopsis thaliana* ubiquinone polyprenyltransferase, AtPPT1. Nodes with IQ-TREE ultrafast parametric support values >0.95 are indicated by gray circles on the preceding branch. The branches and outer color bar are color-coded to match the taxonomic classification of each sequence. *Lithospermum erythrorhizon* sequences are indicated by the red font. The hypothetical location of the inferred duplication via retrotransposition in the ancestor of genes encoding PGTs and PGT-like homologs is indicated by the gray arrow. **b** Heatmap showing the gene expression pattern of *L. erythrorhizon* prenyltransferase genes in whole roots (R), aerial tissue (A), root periderm (P), root vascular (V), hairy root grown in the dark (D), and hairy root grown in the light (L). Conditions where shikonin is most abundant are indicated with an aterisk (*). The cladogram (left) shows the evolutionary relationship between prenyltransferase genes according to the overall maximum likelihood phylogeny in part a

To better understand the evolutionary history of LePGT1 and LePGT2, we constructed a robust phylogenetic tree of OG0000509 prenyltransferases (Fig. 3a). Of the 13 sequences in *L. erythrorhizon*, one was excluded from the phylogenetic analysis due to it being a suspected pseudogene (Leryth_015069), which is located ~10 kb away from *LePGT1* and is likely the result of a tandem duplication event (Fig. S2). Pseudogenization is suspected due to the fact that the sequence appears to be truncated and is missing the conserved NDXXD motif indicative of putative prenyl diphosphate binding (Fig. S3)^32^. In agreement with the OrthoFinder analysis, the phylogeny shows a large radiation of prenyltransferase genes in the Boraginales followed by an additional radiation in the Boraginaceae. The Leryth_011786 gene copy is notable in that it is on a small branch relative to the other *L. erythrorhizon* sequences and groups closest to homologs in the other lamiids (Fig. 3a), suggesting it is likely the “missing” ubiquinone prenyltransferase^38^ (hereafter referred to LePPT1).

As recognized by Kusano et al.^38^, *LePGT1* and *LePGT2* are both comprised of a single exon (Fig. S2). The six *L. erythrorhizon* genes most closely related to *LePGT1* and *LePGT2* (Leryth_008226, Leryth_027669, Leryth_027448, Leryth_000057, Leryth_020476, Leryth_003298) are also single exonic. In contrast, *AtPPT1*, as well as its predicted *L. erythrorhizon* ortholog, *LePPT1*, are multi-exonic, each containing eight exons. The three remaining unclassified *L. erythrorhizon* genes in the phylogeny were found to contain a variable number of exons: Leryth_023194 with ten exons, Leryth_011510 with eight exons, and Leryth_006101 with ten exons. The loss of exons in *LePGT1* and *LePGT2*, and the six additional *PGT-like* genes, suggests that a retrotranspotition event is responsible for the duplication that gave rise to the specialized prenyltransferase genes involved in the production of shikonin.

In order to investigate the mechanisms and timing of the PGT gene family expansion, we identified syntenic blocks for the entire *L. erythrorhizon* genome assembly and performed synonymous substitution (Ks) pattern analyses. Regions of shared Ks values among homologous genes within syntenic blocks would be indicative of a possible whole-genome duplication (WGD) in the ancestor of *L. erythrorhizon*. We identified a (Ks) peak at roughly ~0.45, a value that is suggestive of a potential polyploidy event^39^ (Fig. S4a), and roughly matches the results of Tang et al., who performed a larger analysis of WGD in the Boraginaceae. In the Tang analysis, the Ks peak of ~0.417 was proposed to have arisen via a WGD in the ancestor of the Boraginaceae roughly 25 MYA^20^. Given evidence of a probable WGD in the ancestor of *L*. erythrorrhizon, the expansion of the OG0000509 genes could have arisen due to the WGD or subsequent duplication processes (e.g., tandem duplication, retrotransposition). However, only two syntenic blocks containing PGT and PGT-like genes were identified (Table S10). The first syntenic block had a median Ks of 0.534 and contained PGT1 (Leryth_015168) and another single exonic PGT-like gene (Leryth_008226) (Fig. S4b). The second syntenic block had a median Ks of 0.466 and contained two multi-exonic PGT-like genes (Leryth_011510 and Leryth_006101) (Fig. S4c). Given that the median Ks of the two syntenic blocks containing PGT and PGT-like genes are approximate to the multi-locus peak (~0.45) in the Ks distribution (Fig. S4a), it is possible that these duplications arose via the WGD event. Though, the fact that there is shared synteny between two multi-exonic homologs as well as between two single-exonic homologs, while no evidence of shared synteny between multi-exonic homologs and single-exonic homologs was observed, suggests that the putative retrotransposition event occurred prior to the whole-genome duplication. This apparent lack of synteny also indicates that the contigs harboring PGT homologs are not simply haplotypes resulting from the genome assembly process, but that these genes are found on contigs with unique neighboring genetic sequence.

Aside from the suspected pseudogene, all other encoded prenyltransferases in orthogroup OG0000509 contain the conserved NDXXD motif indicative of putative prenyl diphosphate binding (Fig. S3)^32^. All but two unclassified homologs (Leryth_023194 and Leryth_006101) also maintained the GX(K/Y)STAL sequence motif conserved in this subfamily of 4HB:prenyltransferases (Fig. S3)^32^. One of these unclassified homologs, Leryth_023194, also contained an N-terminal chloroplast targeting sequence (Table S11). None of the other prenyltransferase homologs contained a detectable signal or transit peptide. Lastly, we checked the relative gene expression of all prenyltransferase homologs in three tissue/growth condition comparisons in which shikonin levels and/or expression of shikonin pathway genes are variably abundant: *L. erythrorhizon* whole root tissue versus aboveground tissue^37^, root outer periderm versus inner vascular tissue (Fig. S5), and hairy root tissue cultures grown in M9 in the dark versus in B5 in the light^40^. *LePGT1 and LePGT2*, along with *PGT-like* homologs Leryth_027448 and Leryth_027669 were significantly overrepresented (adjusted *p* value < 0.05, log_2_-fold change >1) in conditions associated with known increased shikonin production or shikonin pathway gene expression states (whole root tissue, root periderm tissue, and hairy root grown in M9 in the dark; Fig. 3b; Table S9). Additionally, *PGT-like* homolog Leryth_020476 was significantly overrepresented in whole root and root periderm tissue (Fig. 3b; Table S9). Lastly, Leryth_000057 and Leyrth_023194 were significantly overrepresented in root periderm tissue. The remaining PGT homologs showed zero to low expression in all samples.

### LePGT1 is the predominant PGT functioning in the shikonin pathway

**Fig. 4:**
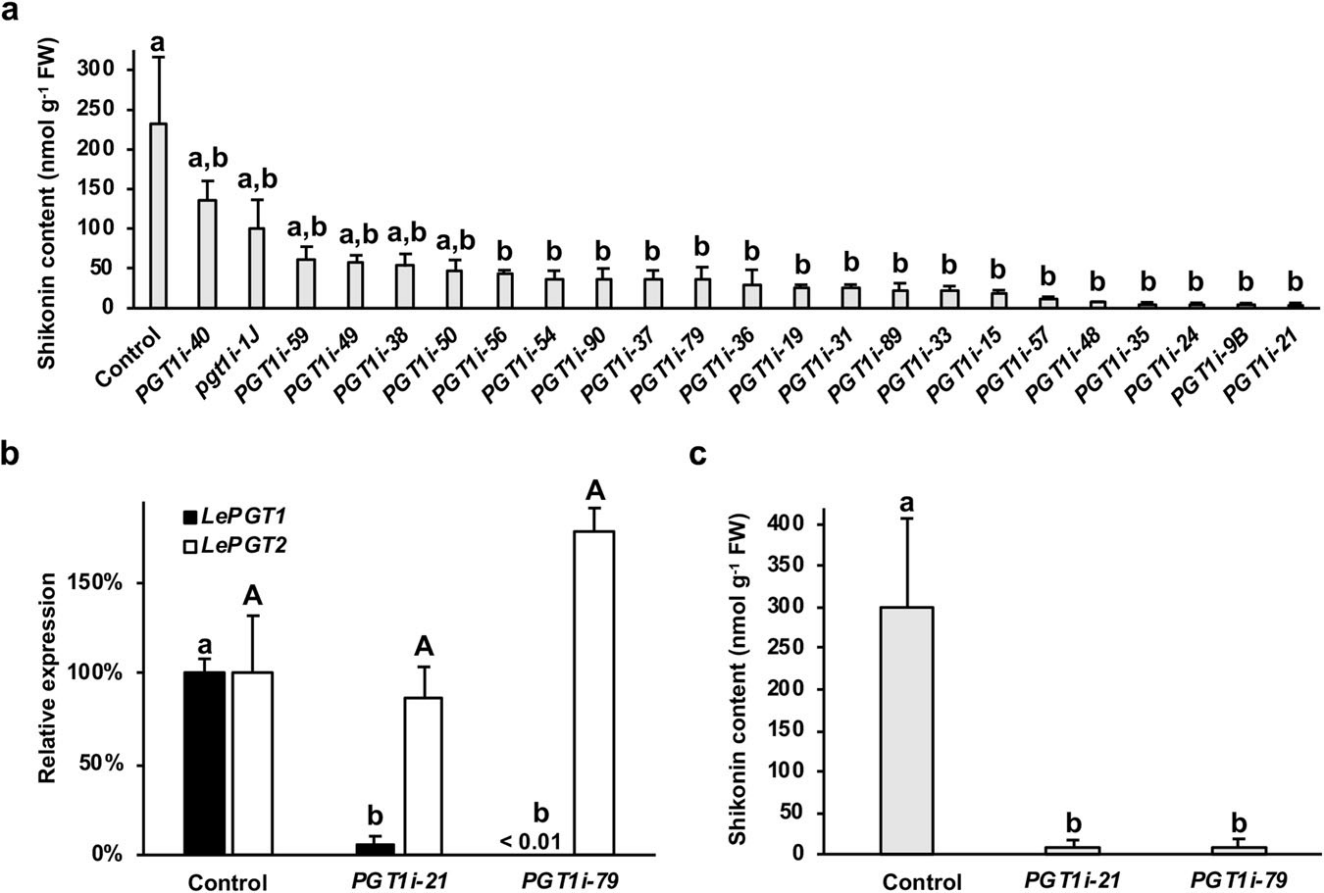
In vivo characterization of LePGT1. **a** Screening of *LePGT1*-RNAi (*PGT1i*) lines based on total shikonin levels present in liquid culture media 3 d after transfer of 14-d-old hairy roots to M9 and darkness. **b** Expression levels of *LePGT1* and *LePGT2* in hairy roots of two independent *PGT1i* lines compared to control. **c** Analysis of total shikonin in same lines used to measure expression in panel **b**. All data are means ± SEM (*n* = 3–4 biological replicates). Different letters indicate significant differences via analysis of variance (ANOVA) followed by post-hoc Tukey test (α = 0.05). In **b**, lowercase and capital letters correspond to statistical comparisons for *LePGT1* and *LePGT2* expression, respectively

LePGT1 is considered as the key regulatory enzyme in the shikonin pathway^41,42^. As there are no reported genetic studies with *PGTs*, and in light of the newly identified PGT-like encoding genes found in the *L. erythrorhizon* genome (Fig. 4), we knocked down *LePGT1* expression in *L. erythrorhizon* hairy roots to investigate if LePGT1 is indeed the predominant PGT controlling shikonin production. Several independent *PGT1*-RNAi (*PGT1i*) lines were generated, excised, transferred to B5 media plates for selection, and then screened based on total shikonins (the sum of shikonin plus its derivatives) production in liquid culture using HPLC coupled with diode array detection (DAD). Individual lines were cultured in liquid B5 in constant light without selection for 14 d and then transferred to M9 and constant darkness to induce shikonin production. Analysis of culture media 3 d after transfer to M9 and darkness revealed 17 *PGT1i* lines producing between 1 and 59% of the total shikonins synthesized by control hairy roots sampled at the same time (Fig. 4a). Two lines, *PGT1i*-21 and *PGT1i*-79, were further analyzed by qRT-PCR. Both lines were found to have greater than 95% reduced *LePGT1* levels while expression of *LePGT2* remained statistically unchanged compared to the control (Fig. 4b). Because the gene encoding LePGT1 is more similar to LePGT2 than any of the PGT-likes (Fig. 3a), these data indicate that the RNAi construct specifically targeted *LePGT1*. Analysis of the culture media from the same hairy roots used to perform qRT-PCR revealed that the reduction in *LePGT1* expression (Fig. 4b) correlated with a more than 95% decrease in total shikonin content (Fig. 4c). These results provide further support for LePGT1 being predominantly responsible for the formation of 3-geranyl-4HBA. They also imply that the PGT-like proteins encoded in the *L. erythrorhizon* genome likely do not play a major role in shikonin production.

## Discussion

In this study, we report the first de novo assembly of a *L. erythrorhizon* genome obtained through a combination of ONT long-read and Illumina HiSeq short-read sequencing technologies. The ~367.41 Mb assembly contained 27,720 predicted protein-coding genes that were clustered into 14,885 orthologous gene families, 79.84% of which were also found in other species in our comparative phylogenetic analysis (Fig. 2). A significant number of these orthogroups (2818) appear to have duplicated in the last common ancestor of the five Boraginaceae species in our analysis, and another 3908 orthogroups appear to have duplicated in the last common ancestor of the four known shikonin/alkannin-producing species. Among the 6726 orthogroups duplicated at these internodes is OG0000509, the orthogroup containing *AtPPT1* and *LePPT1* (genes involved in the primary metabolic process of ubiquinone biosynthesis) as well as the specialized genes *LePGT1* and *LePGT2* involved in shikonin biosynthesis. An additional nine homologous prenyltransferases of unknown function were also identified in the *L. erythrorhizon* genome. This analysis illustrates the importance of gene duplication in the evolution of the shikonin/alkannin pathway, in agreement with other analyses of specialized metabolism in plants (e.g.,^43–45^). In the case of the *PGT* and *PGT-like* sequences in *L. erythrorhizon*, the initial duplication event appears to have occurred via retrotransposition based on the fact that these sequences appear to have lost all introns (Fig. 3a; Table S9). The relative contribution of retrotransposition compared to other processes that generate gene duplicates (e.g., whole-genome duplication) to metabolic innovation in plants remains to be investigated. Overall, this genomic resource will complement the available *E. plantagineum* genome^20^ and the extensive sets of transcriptomes (e.g., refs ^14–16^) and proteomes^17^ published from Boraginaceae species for elucidating the shikonin/alkannin pathway and its evolutionary origin, as well as the evolution of specialized metabolism more generally in this family.

**Fig. 5:**
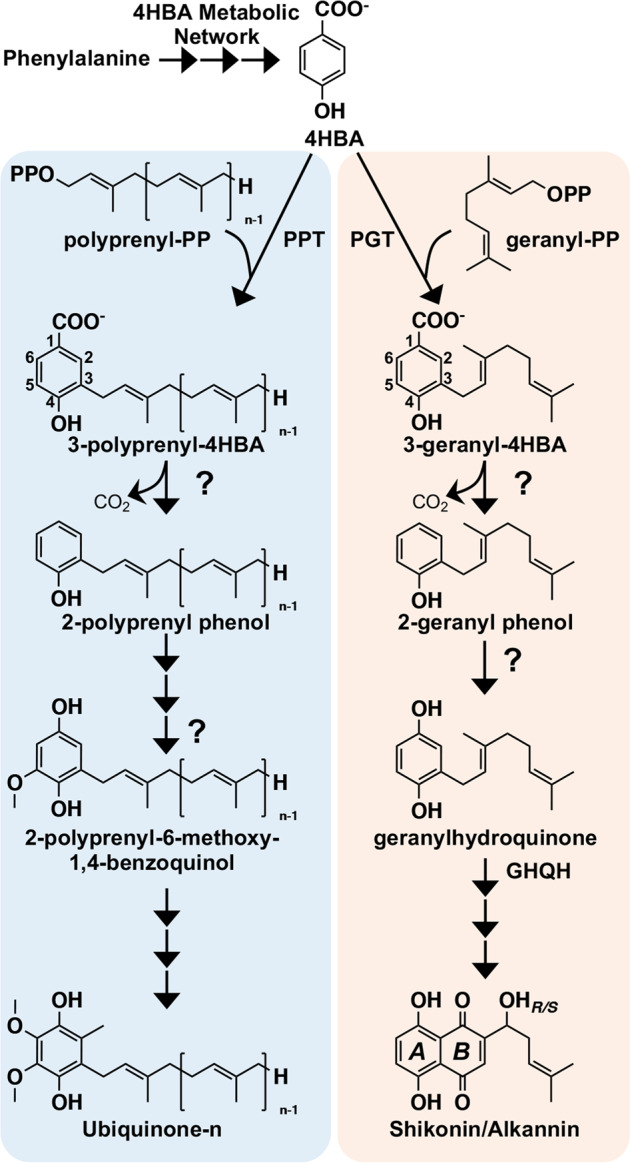
Similarities between shikonin and ubiquinone biosynthesis. The committed steps of the shikonin and ubiquinone pathways rely on homologous prenyltransferases that conjugate 4-hydroxybenzoic acid (4HBA), derived from phenylalanine, with a prenyl diphosphate precursor. Subsequent decarboxylation and hydroxylation at the C1 position of the 3-prenylated/geranylated 4HBA ring is required in both pathways, although the responsible enzymes (depicted by “?”) in each route remain unknown. Non-oxidative decarboxylation of the prenylated-4HBA ring is shown as it occurs in bacteria. It is possible that plants use an oxidative decarboxylation mechanism, which would result in concomitant decarboxylation and hydroxylation at the C1 position, bypassing the phenolic intermediates. Abbreviations: GHQH, geranylhydroquinone hydroxylase; PGT, *p*-hydroxybenzoate:geranyltransferase; PPT, polyprenyltransferase

The connection between shikonin/alkannin and ubiquinone biosynthesis is not limited to homologous prenyltransferases. The hydroxybenzene ring, ring *A* (Fig. 5), of shikonin/alkannin’s naphthazarin moiety is derived from L-phenylalanine via cinnamic acid and 4HBA^4,46^–the same route that is partially responsible for forming the benzoquinone ring of ubiquinone (coenzyme Q) in plants^47,48^. Like the shikonin/alkannin pathway, ubiquinone biosynthesis starts off with the conjugation of 4HBA with a polyprenyldiphosphate, catalyzed by a PPT (Fig. 5). In addition to the analogous PPT- and PGT-catalyzed reactions, the ubiquinone and shikonin/alkannin pathways share other similar ring modification reactions that occur early in their respective pathways (Fig. 5). Both ubiquinone and shikonin/alkannin biosynthesis require the prenylated 4HBA ring to be decarboxylated and hydroxylated at the C1 position, though the sequence of reactions and necessary enzymes are unknown in both pathways. In the *Escherichia coli* ubiquinone pathway, the non-oxidative ring decarboxylation of 3-polyprenyl-4HBA is catalyzed by UbiD in concert with a UbiX chaperone for substrate reorientation^49^. Orthologs of genes encoding UbiD and UbiX are absent from plant genomes. Therefore, if plants also use a series of non-oxidative decarboxylation and hydroxylation steps to modify the C1 position of the 4HBA ring, they do so using other evolved enzymes. It is also possible that plants have evolved to achieve the decarboxylation and hydroxylation via an oxidative decarboxylase that would carry out both reactions. Whatever the mechanism for C1 decarboxylation and hydroxylation, if it is indeed shared between shikonin/alkannin and ubiquinone biosynthesis, it is possible that there is an evolutionary linkage among the enzymes involved given the other metabolic connections between the two pathways.

Downregulation of *LePGT1* expression by 95% was sufficient to reduce shikonin production by more than 97% 3 d after transfer of hairy roots to M9 media and darkness (Fig. 4b, c). While this provides strong evidence that LePGT1 is principally controlling shikonin formation, it does not rule out that LePGT2 still plays a significant role. Like *LePGT1*, *LePGT2* is highly expressed under shikonin producing conditions (Fig. 3b; Table S9)^32,50^. One explanation for achieving near abolishment of shikonin by only knocking down *LePGT1* could be that LePGT1 and LePGT2 form heteromers in vivo. Biochemical studies, however, suggest that LePGT1 is capable of functioning homomericly^41,42^, so this appears unlikely. Another possibility is that LePGT1 is the predominant form functioning early in shikonin-producing conditions because of its biochemical properties. LePGT1 has 5- and 10-fold higher affinities for 4HBA and GPP, respectively, compared to LePGT2^32^. It can be envisioned that early in hairy root culture establishment, when precursor concentrations are a priori low, that LePGT1 is the de facto PGT responsible for forming 3-geranyl-4HBA. In this study, shikonin levels were measured at day 3, the first day that visible production occurred in control cultures. It is possible that over time, as precursor pools increase, the relative contribution of LePGT2 would become greater. If any of the PGT-like proteins encoded in the *L. erythrorhizon* genome also contribute PGT activity, their relative contributions would also increase over the culture period. In light of the current study, reports of multiple *PGT* unigenes and their variable expression patterns in other transcriptomic studies^19,37^ should be re-investigated to determine if any encode PGT-like proteins. More detailed investigations looking at shikonin formation over time using metabolic flux analysis with stable isotopic labeling are needed to determine the temporal contributions of PGTs.

## Conclusions

The first genome assembly from the medicinally and economically important plant *L. erythrorhizon* is expected to advance understanding of the evolutionary history of the Boraginales. As just the second genome from a member of this order, the other from *E. plantagineum*^20^, the *L. erythrorhizon* genome will provide another piece of the puzzle needed to reconstruct the phylogenetic relationships among the Boraginales, Solanales, Gentianales, and Laminales (Fig. 2). The *L. erythrorhizon* genome will also serve as a novel tool for elucidating the remaining missing steps in the shikonin/alkannin pathway and for filling gaps in knowledge about its metabolic origin. Our phylogenetic analysis of prenyltransferases encoded in the *L. erythrorhizon* genome (Fig. 3a) has already led to the remarkable discovery of several additional encoded PGTs. It also provided evidence to suggest that *PGTs* arose in a common ancestor of modern shikonin/alkannin-producing Boraginaceae species via a retrotransposition-derived duplication event and subsequent neofunctionalization of an ancestral prenyltransferase gene. Based on homology between PGTs and PPTs it is possible that this points to an evolutionary link between ubiquinone and shikonin/alkannin biosynthesis, especially considering the other metabolic similarities shared between the two pathways (Fig. 5). This would not be the first connection found between primary and specialized quinone metabolism in plants as it was recently reported that the pathway to synthesize the naphthoquinone moiety of juglone in black walnut trees (*Juglans nigra*) is shared with the phylloquinone (vitamin K1) pathway^51^. Taken together, the results from our study provide several new leads for investigating the evolution of specialized metabolism in the Boraginaceae.

## Materials and methods

### Plant materials, growth conditions, and general experimental procedures

Seeds of *L. erythrorhizon* (accession Siebold & Zucc.) were obtained from the seed bank at the Leibniz Institute of Plant Genetics and Crop Plant Research (IPK) in Germany and plants were propagated under standard greenhouse conditions to bulk seeds.

For all hairy root culture work, *L. erythrorhizon* Siebold & Zucc. seeds were sterilized by chlorine gas according to Lindsey et al.^52^. After exposure to chlorine gas for 2 h, seeds were rinsed five times with sterile water and shaken in 200 µg/mL cefotaxime overnight. They were then rinsed with sterile water and plated on half-strength Murashige and Skoog (MS, Phytotech Labs) media with 0.1% Plant Preservation Mixture (PPM^TM^, Plant Cell Technology). Seeds were stratified at 4 °C for 2 weeks and moved to room temperature under 12:12 light:dark cycle. Once germinated, seed coats were removed, and each seedling was transferred to a magenta box with half-strength MS media. Plants with their second pairs of true leaves were used in hairy root transformation.

Hairy roots were maintained in solid Gamborg B5 media (Phytotech Labs) containing 3% sucrose and 10 mg/L Basta (PlantMedia^TM^). Hairy roots were subcultured every two weeks. For quantification of shikonin, 1-cm hairy root fragments were transferred to 20 mL liquid Gamborg B5 media and grown under constant light at 28 °C for two weeks without Basta. Shikonin production was then induced by transferring hairy roots to M9 media^53^ and culturing at 28 °C in constant darkness without Basta.

### Nanopore sequencing

For nanopore sequencing, leaves from an in vitro cultured 3-month-old *L. erythrorhizon* Siebold & Zucc. plant were frozen in liquid nitrogen, ground by mortar and pestle, and high molecular weight genomic DNA was extracted using a CTAB phenol chloroform extraction protocol (available on protocols.io: 10.17504/protocols.io.bamnic5e) and purified using a Genomic DNA Clean and Concentrator kit (Zymo Research). At least 2 μg of gDNA was used as input for an Oxford Nanopore LSK-109 library ligation kit and sequenced on R9 MinION flow cells. Base calling was performed with Guppy v2.3.5^54^. Reads less than 3 kilobase pairs long or with quality scores less than 7 were discarded. Reads are available for download at the NCBI Sequence Read Archive (PRJNA596998).

### Genome assembly

*Lithospermum erythrorhizon* Illumina gDNA PE reads sequenced by Nanjing University in 2018 from an unknown accession were downloaded from NCBI SRA database experiment SRR5644206 and assembled with Abyss v2.1.5^55^ using a k-mer size of 75. The assembled Abyss contigs and the ONT long reads served as input for the DBG2OLC hybrid assembler using the following parameters: KmerCovTh 2, AdaptiveTh 0.0001, MinOverlap 20, RemoveChimera 1, k 17^21^. The resulting hybrid assembly was error corrected via five rounds of polishing with the Illumina gDNA reads using Pilon v1.23^56^. Five additional rounds of Pilon polishing were performed using *L. erythrorhizon* Siebold & Zucc. stranded RNA-seq reads (see RNA-seq experiments section below) as input to fix single nucleotide errors in transcribed regions. Genome size was estimated using GenomeScope^57^ with a k-mer (k = 21) depth distribution of the Illumina gDNA reads calculated using Jellyfish v2.2.10^58^. Illumina gDNA reads were mapped back to the final assembly using BWA v0.7.15^59^, RNAseq reads were mapped using STAR v2.5.4^60^, and ONT reads were mapped using minimap2 v2.13^61^. Histograms of Illumina and ONT read depth when mapped to the final assembly were generated by the program purge_haplotigs v1.1.1 to assess the level of assembly heterozygosity^62^.

### Genome annotation

De novo repeat identification was performed using RepeatModeler v1.0.9 and masked using RepeatMasker v4.0.7 (http://repeatmasker.org). Gene model and protein prediction was conducted with MAKER2 v2.31.10^63^ by supplying protein homology-based evidence, transcriptomic evidence in the form of a genome guided transcriptome assembly generated from in-house Siebold & Zucc. stranded RNA-seq reads (see RNA-seq experiments section below) using Trinity v2.5.1^64^, and ab initio gene calling using SNAP^65^ and BRAKER2^66^. Gene models with an AED score <0.9 or ones that encoded a predicted protein <30 amino acids long were excluded from the final gene set. Additional information on the full annotation pipeline is presented in Fig. S6. Conservation of core genes was performed using BUSCO v2.0^26^. Functional annotation of the final protein set was performed using InterProScan^22^ and TargetP^67^.

We checked the genome for possible contamination using the Alien Index (AI) pipeline (https://github.rcac.purdue.edu/jwisecav/phylo-pipe; last updated August 26, 2019) as previously described^27^. Briefly, each predicted protein sequence was queried against the NCBI RefSeq database (release 97) using Diamond v0.9.22.123^68^, and the AI score was calculated based on the output. The AI score is given by the formula: AI = nbsO-nbsE, where *nbsO* is the normalized bit score of the best hit to a species outside of the eudicot lineage, *nbsE* is the normalized bit score of the best hit to a species within the eudicot lineage (skipping all hits to *L. erythrorhizon* present in the RefSeq database). AI scores range from -1 to 1, being greater than zero if the predicted protein sequence had a better hit to a non-eudicot species, suggestive of either HGT or contamination^27^.

### RNA-seq experiments

For the *L. erythrorhizon* root periderm and vascular tissues RNA-seq experiment, 3-month-old Siebold & Zucc. plants grown in soil under standard greenhouse conditions were harvested. Roots were collected from nine individual plants and divided into three groups, each containing three unique individuals. The periderm and vascular tissues were isolated by peeling the periderm from the roots (Fig. S5a), and the prepared portions from the three individuals in each group were pooled. Tissues were frozen in liquid nitrogen, ground by mortar and pestle, and 100 mg was used to analyze total shikonin content each sample (Fig. S5b). From the same sets of samples, RNA was extracted as described below, quantified, and DNase-treated (NEB) according to the manufacturer’s instructions. A total of six cDNA libraries from the three biological replicates prepared from each of the *L. erythrorhizon* periderm and vascular tissue pools, were constructed using a ribominus TruSeq Stranded Total RNA library prep kit (Illumina, San Diego, CA), and 101-bp paired-end reads were generated via Illumina HiSeq 2500 at the Purdue Genomics Center, with at least 67 million reads per library. Sequence quality was assessed by FastQC (v. 0.10.0; http://www.bioinformatics.babraham.ac.uk). The raw data were submitted to the Sequence Read Archive (http://www.ncbi.nlm.nih.gov/sra/) and are available at the NCBI Sequence Read Archive (PRJNA596998).

The experimental design for the RNA-seq experiment comparing *L. erythrorhizon* hairy roots sampled in B5 in the light and M9 in the dark was based on a previous report of observed rapid increases in expression of shikonin precursor pathway genes, and in *PGT*, within 2 h after switching *L. erythrorhizon* cell cultures from growth in B5 in the light to growth in M9 in darkness^40^. In this study, several cultures from three independently generated *L. erythrorhizon* hairy root lines were started in liquid Gamborg B5 media containing 3% sucrose at 28 °C in the light (~100 µE m^−2^ s^−1^). After 2 weeks, hairy roots from three cultures for each of the three lines (*n* = 3 biological replicates per line) were harvested and pooled to represent the B5 light-treated samples. The remaining hairy root cultures were transferred to M9 media and darkness. After 2 h, hairy roots from three cultures for each of the three lines (*n* = 3 biological replicates per line) were harvested and pooled to represent the M9 dark-treated samples. Samples were frozen in liquid nitrogen, ground by mortar and pestle, and RNA was extracted as described below. Six cDNA libraries were generated with a TruSeq Stranded mRNA library prep kit (Illumina, San Diego, CA) and were sequenced on an Illumina NovaSeq 6000 at the Purdue Genomics Center. Sequence quality assessment were performed as described above for the periderm and vascular tissues RNA-seq experiment. The raw data were submitted to the Sequence Read Archive (http://www.ncbi.nlm.nih.gov/sra/) and are available at the NCBI Sequence Read Archive (PRJNA596998).

Additionally, unstranded RNA-seq data of *L. erythrorhizon* whole roots and aerial tissue from an unknown accession was downloaded from the NCBI SRA (experiments SRR3957230 and SRR3957231) to include in the gene expression analysis. Gene abundance estimates of PGT and PGT-like genes (Fig. 3b, Table S9) were measured using Kallisto v0.45.0^69^ and normalized for library depth using DESeq2^70^. Differential expression status was determined using the EdgeR v3.24.3^71^ package. For the EdgeR analysis, raw counts were normalized into effective library sizes using the trimmed mean of M-values (TMM) method^72^, and exact tests were conducted using a trended dispersion value and a double tail reject region. A false discovery rate was calculated using the Benjamini–Hochberg procedure^73^. Genes with a log_2_-fold change in abundance greater than 1 and false discovery rate less than 0.05 were considered as differentially represented.

### De novo transcriptome assemblies of additional Boraginaceae species

Illumina RNA-seq reads from additional shikonin-producing species *L. officinale*, *Arnebia euchroma* and *Echium plantagineum*^19,37^ were downloaded from the following NCBI SRA experiments: SRR4034889, SRR4034892, SRR4034890, SRR4034891, SRR6799516, SRR6799517, and SRR6799518. Raw RNA-seq reads were normalized and error corrected with BBnorm using a target size of 40 and a minimum depth of 2 (software last modified October 19, 2017) and Tadpole using default parameters (software last modified June 27, 2017), programs from the BBMap software package^74^. The resulting clean reads were assembled de novo using Trinity v2.5.1^64^ with default parameters for stranded (*L. officinale*) and unstranded (*E. plantagineum* and *A. euchroma*) libraries. Coding regions were inferred using TransDecoder v3.0.1^75^.

### Identification of orthologous gene families

Homology between the predicted proteomes of *L. erythrorhizon* and 31 other eudicot species was determined with OrthoFinder v2.1.2 using the following parameters: -S diamond -M msa -T fasttree^28^. The species tree in Fig. 2 was generated using TreeGraph2 v2.15.0^76^. Hypergeometric tests were performed in python using the SciPy library hypergeom, and *p*-values were adjusted for multiple comparisons using the StatsModels library multitest with the Benjamini & Hochberg (BH) method^73^.

### Phylogenetic analysis

We performed a separate phylogenetic analysis of the orthogroup containing LePGT1 and LePGT2 (OG0000509). Three sequences were excluded due to their long branches, including the suspected pseudogene Leryth_015069. The remaining sequences were aligned with MAFFT v7.407 using the E-INS-I strategy and following parameters: --maxiterate 1000 --bl 45 --op 1.0 --retree 3^77^. The maximum likelihood phylogeny was constructed using IQ-TREE version 1.6.10^78^ using the built in ModelFinder to determine the best-fit substitution model^79^ and performing SH-aLRT and ultrafast bootstrapping analyses with 1,000 replicates each. The gene tree in Fig. 3a was generated using iTOL v4^80^.

### Synteny analysis

Regions of shared synteny within the genome of *L. erythrorhizon* were detected using SynMap2 on the online Comparative Genomics Platform (CoGe) using default settings with the exception that the merge syntenic blocks algorithm was set to Quota Align Merge, the syntenic depth algorithm was set to Quota Align, and the CodeML option was activated to calculate substitution rates between syntenic CDS pairs. For syntenic blocks containing PGT genes and their homologs, the encompasing contigs were aligned using promer (v3.07) of the MUMmer4 alignment system^81^.

### Cloning and generation of LePGT1i hairy root lines

For the *LePGT1*-RNAi construct, ﻿DNA containing two spliced *LePGT1* cDNA fragments of the coding region corresponding to nucleotides 179-698 and 179-502, the latter in antisense orientation to create a hairpin structure, was synthesized (Genscript, Piscataway, NJ). 5’-CACC was added for subcloning into ﻿pENTR^TM^/D-TOPO (Invitrogen^TM^, Carlsbad, CA), sequence verified, and transferred into the destination vector, pB2GW7^82^, by recombination using LR Clonase Enzyme Mix^TM^ (Invitrogen). The final construct, pB2GW7*-PGT1i*, was transformed into *Agrobacterium rhizogenes* strain ATCC 15834 competent cells by freeze-thaw transformation^83^. Briefly, competent cells were incubated with 100 ng of pB2GW7-*PGT1i* for 15 min on ice. Then, cells were snap frozen in liquid nitrogen for 5 min and consecutively thawed at 37 °C for 5 min. Nutrient Broth (NB) media was added to the culture and kept in 37 °C with shaking for 2 h before being plated on NB agar containing 50 µg/mL spectinomycin for selection.

*L. erythrorhizon* hairy root *PGT1i* lines were generated based on the protocol from Fang et al.^84^ with slight modification. *A. rhizogenes* containing pB2GW7*-PGT1i* was inoculated in NB with 50 µg/mL spectinomycin and kept in shaking incubator at 28 °C until reached OD_600_ = 1. Then, acetosyringone was added to the media to a final concentration of 0.1 mM and the culture was grown further for 4 h in dark. The *A. rhizogenes* was then pelleted by centrifugation. The pellet was washed and resuspended in half-strength MS containing 0.1 mM acetosyringone. Each stem of sterile plants grown in tissue culture was wounded by surgical blade and the prepared culture was applied to the wounded area by cotton swab. The plants were then kept in darkness for 1 d and returned to normal growth conditions. The hairy roots emerged between 10 and 28 d post infection. Emergent roots were excised and placed on Gamborg B5 media with 3% sucrose and 200 µg/mL cefotaxime to eliminate *A*. *rhizogenes*. After 2 weeks, hairy roots were transferred to Gamborg B5 media containing 3% sucrose and 10 mg/L Basta for selection for 2 weeks. Hairy root lines transformed by *A. rhizogenes* without pB2GW7-*PGT1i* were generated to use as control.

### RNA extraction and qRT-PCR

Total RNA was extracted from ~20 mg of hairy root tissues according to the protocol from Ghawana et al.^85^ Briefly, the samples were frozen by liquid nitrogen and ground by mortar and pestle. 2 mL of RNA extraction buffer (phenol containing 0.1% sodium dodecyl sulfate, 0.32 M sodium acetate, and 0.1 M ethylenediaminetetra acetic acid) was added to the sample in the mortar and mixed, followed by addition 0.8 mL of RNAse-free water. After mixing, the mixture was incubated for 5 min before transfer to microtubes. 0.3 mL of chloroform was added, and the sample was vortexed and centrifuged at 4 °C, 13,000 rpm for 10 min. The supernatant was then transferred to the new tube containing 0.6 mL isopropanol. Next, the sample was mixed by inverting, and nucleic acids were precipitated at −20 °C for 10 min. After precipitation, the sample was centrifuged at 4 °C for 10 min. The pellet was washed with 70% ethanol before air drying. The RNA pellet was dissolved in RNase-free water. The total RNA was concentrated and purified using an RNA Clean & Concentrator Kit (Zymo Research) with on-column DNase treatment (Zymo Research) using the manufacturer protocol. cDNA synthesis was performed by 5X All-In-One RT MasterMix (abm) according to the manufacturer instructions using 500 ng of total RNA.

Expression of *LePGT1* and *LePGT2* was measured by qRT-PCR with comparative quantification using the 2^−ΔΔCT^ method^86^. Primers were designed using Primer-BLAST on NCBI^87^. Due to the sequence similarity of members of the *LePGT* and *LePGT-like* (Table S9) gene family, each primer was checked against all members for possible off-target matches. To minimize off-target amplification, primer pairs were selected that had either (i) four or more mismatches in a primer to all other *LePGT* and *LePGT-like* family genes or (ii) two mismatches in one primer and three mismatches in the other primer to all other *LePGT* and *LePGT-like* family genes^88^. qRT-PCR reactions were performed using a QuantStudio^TM^ 6 (ThermoFisher) in a 10 μL reaction as follows: 5 μL of 5x Fast SYBR Green PCR master mix (ThermoFisher), 1 μL each of the forward and reverse primers (50–900 nM final concentration; Table S12), and 3 μL of diluted cDNA. Expression was normalized to *L. erythrorhizon* glyceraldehyde 3-phosphate dehydrogenase (*LeGAPDH*) using primers from Zhao et al.^89^.

### Shikonin extraction and quantification

The extraction of total shikonins was modified from Boehm et al.^90^. Briefly, a 4 mL sample of growth media from each hairy root line was sampled at day 3 after transfer to M9 and darkness and extracted with 4 mL of chloroform. The chloroform layer was separated and dried under a gentle stream of N_2_ at 40 °C. Base hydrolysis was performed on the remaining residue in the tube by adding 2 mL of 1 M NaOH and shaking for 1 h at room temperature. The solution was neutralized by adding 1 mL of 6 M HCl and vortexed. Shikonin was extracted by adding 3 mL of ethyl acetate by liquid–liquid extraction. The ethyl acetate layer was separated and dried under N_2_, dissolved in 250 µL methanol, and 20 µL was used for detection by high performance liquid chromatography with diode array detection (HPLC-DAD). The extraction procedure was performed in reduced light to minimize the photo-degradation of shikonin.

HPLC-DAD analyses were performed with an Agilent 1260 Infinity HPLC system (Agilent Technologies). Chromatographic separation of shikonin was achieved using a Zorbax SB-C18 column (4.6 × 250 mm, Agilent) kept at 25 °C. The mobile phase gradient started at 60% A (30:70 acetonitrile and water with 0.1% formic acid) and 40% B (30:70 isopropanol and acetonitrile with 0.1% formic acid) with 1 min hold and then linearly increased to 99% B over 15 min with a hold of 4 min, and then returned to 40% B from 19 to 20 min with a hold of 1 min. Shikonin eluted at 8.4 min and was detected at 520 nm by DAD. Instrument operation and data analysis steps were performed through the Agilent ChemStation software. Shikonin quantitation by DAD was done by running a linear range of 1.25, 2.5, 5, 10, and 20 nmol calibration standards, followed by a linear regression formula calculation. Differences between shikonin content in each line (*n* = 3–4 biological replicates) was analyzed using one-way ANOVA and the means were compared with Tukey’s HSD post hoc test at 95% significant level.

For analysis of shikonins from *L. erythrorhizon* root periderm and vascular tissues for RNA-seq, HPLC coupled with fluorescence detection (HPLC-FLD; Fig. S5), chromatographic separation was conducted using a Zorbax Eclipse XDB-C18 column (4.6 × 150 mm, Agilent). The column was eluted at 25 °C using a linear gradient starting from 70% A (water with 0.1% formic acid) and 30% B (acetonitrile with 0.1% formic acid), to 1% A and 99% B over 40 min at a flow rate of 0.5 mL min^−1^, followed by a 10 min re-equilibration step. Shikonin eluted at 25.9 min and was detected by fluorescence using λ_ex_ = 228 nm and λ_em_ = 390 nm after passing ﻿through an in-line post-column dry reactor packed with zinc dust, which was previously used for detection of the 1,4-naphthoquinone juglone^51^.

## Supplementary information

Supplementary Figure 1

Supplementary Figure 2

Supplementary Figure 3

Supplementary Figure 4

Supplementary Figure 5

Supplementary Figure 6

Supplementary Tables

Supplemental Dataset S1

Supplemental Dataset S2

Supplemental Dataset S3

## Data Availability

Raw sequencing reads used for de novo whole-genome assembly and the final genome have been deposited in the Sequence Read Archive database under access number PRJNA596998.
