# Supplementary material for: Hybrid de novo genome assembly of red gromwell (Lithospermum erythrorhizon) reveals evolutionary insight into shikonin biosynthesis
Source: Hortic Res. 2020 Jun 1;7:82. doi: 10.1038/s41438-020-0301-9 (PMC7261806; doi:10.1038/s41438-020-0301-9)
Supplement: Supplementary file 1 — Supplementary Figure 1 [file 41438_2020_301_MOESM1_ESM.pdf]

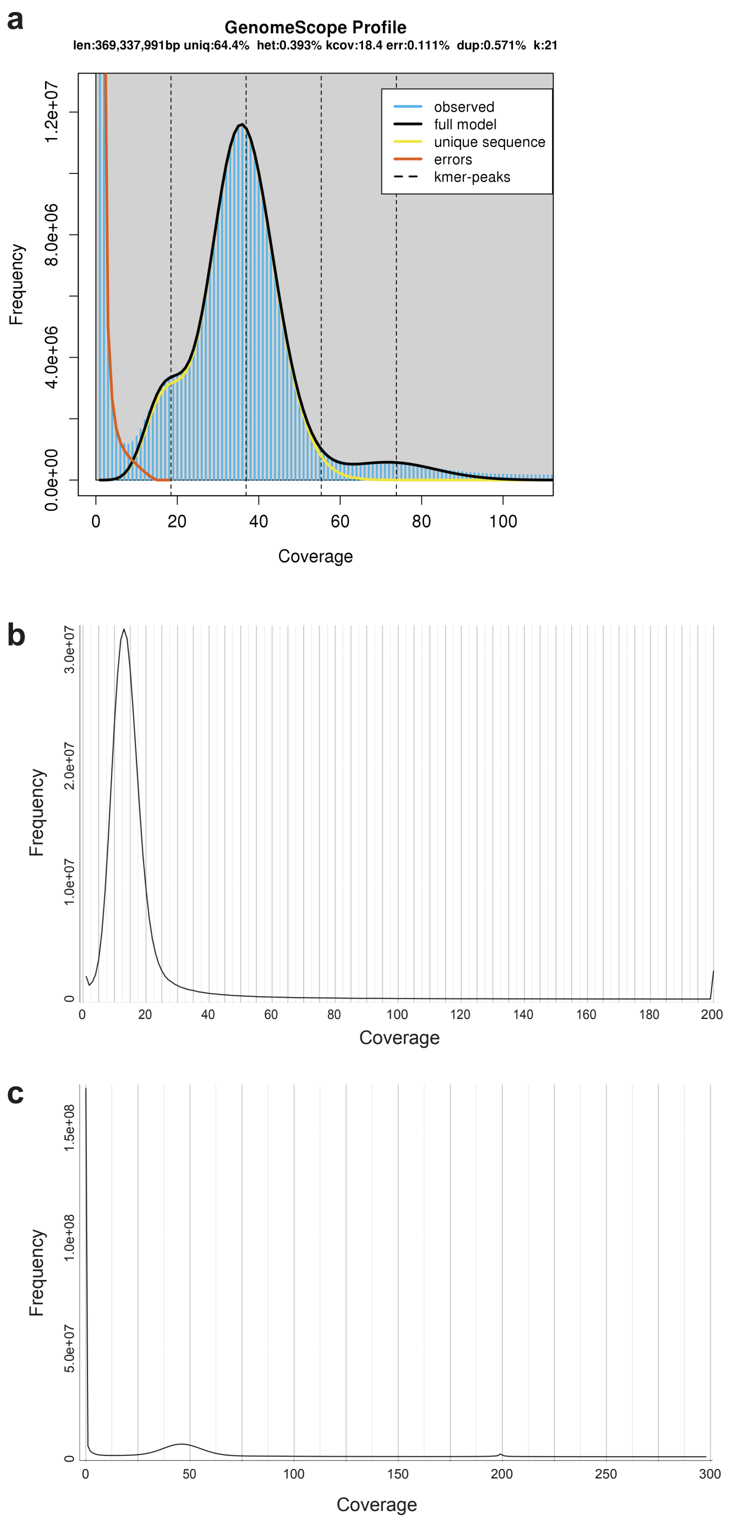

Figure S1. Estimated heterozygosity plots for *L. erythrorhizon*. a) GenomeScope k-mer profile plot of the L. erythrorhizon illumina gDNA reads. The blue bars indicate the observed kmer frequencies, and the black and yellow lines indicate expected distribution of kmers using the GenomeScope model for diploid genomes. b) Purge-haplotigs read-depth histogram of the L. erythrorhizon genome displaying coverage of nanopore reads. c) Purge-haplotigs read-depth histogram of the L. erythrorhizon genome displaying coverage of illumina gDNA reads.
