# Supplementary material for: Hybrid de novo genome assembly of red gromwell (Lithospermum erythrorhizon) reveals evolutionary insight into shikonin biosynthesis
Source: Hortic Res. 2020 Jun 1;7:82. doi: 10.1038/s41438-020-0301-9 (PMC7261806; doi:10.1038/s41438-020-0301-9)
Supplement: Supplementary file 2 — Supplementary Figure 2 [file 41438_2020_301_MOESM2_ESM.pdf]

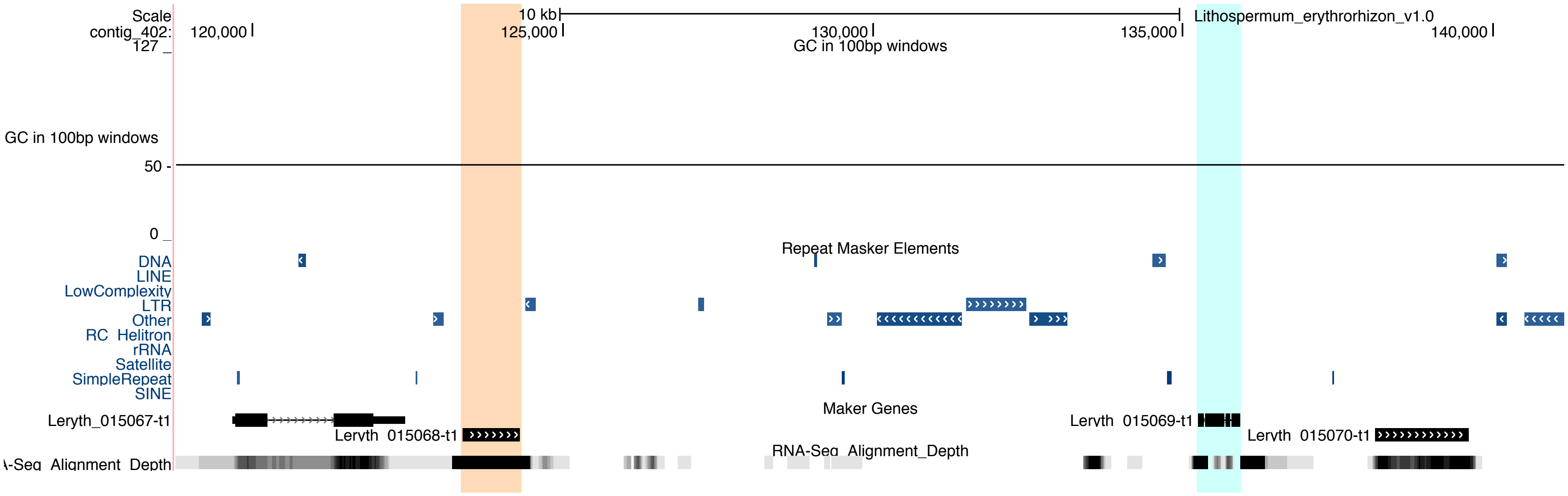

Figure S2a: UCSC Genome Browser region for PGT1 (Leryth_015068). Target gene region is highlighted in orange. Repeat elements are shown in blue. RNAseq alignment depth based on 1kb sliding window. Suspected pseudogene (Leryth_015069) also highlighted in light blue.

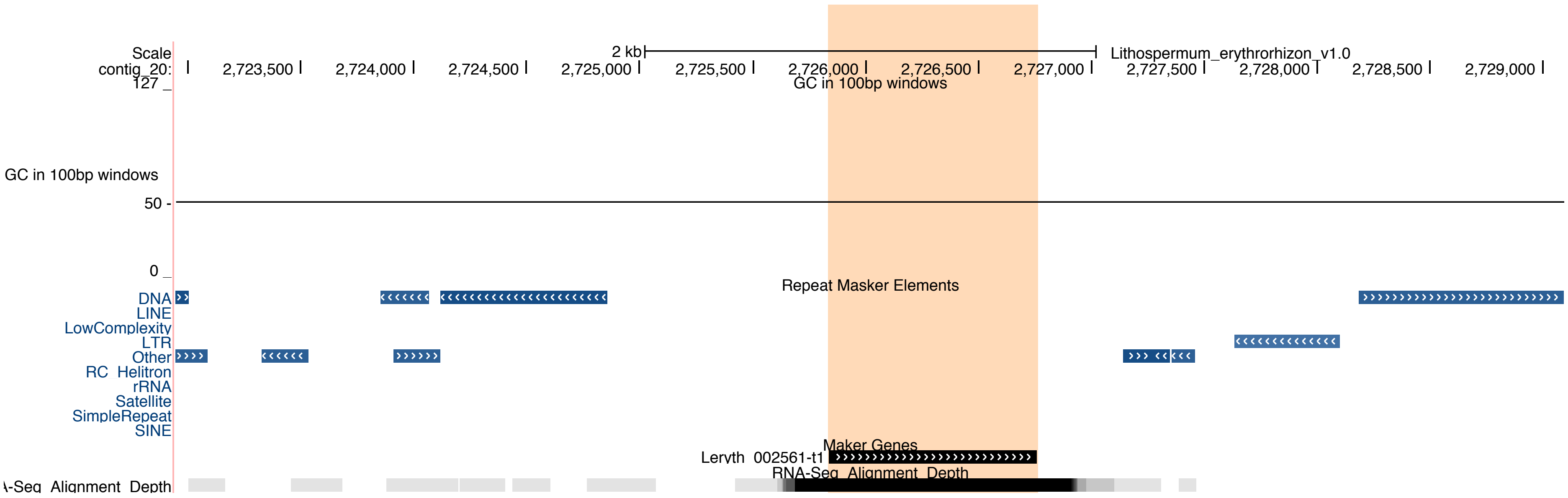

Figure S2b: UCSC Genome Browser region for PGT2 (Leryth_002561). Target gene region is highlighted in orange. Repeat elements are shown in blue. RNAseq alignment depth based on 1kb sliding window.

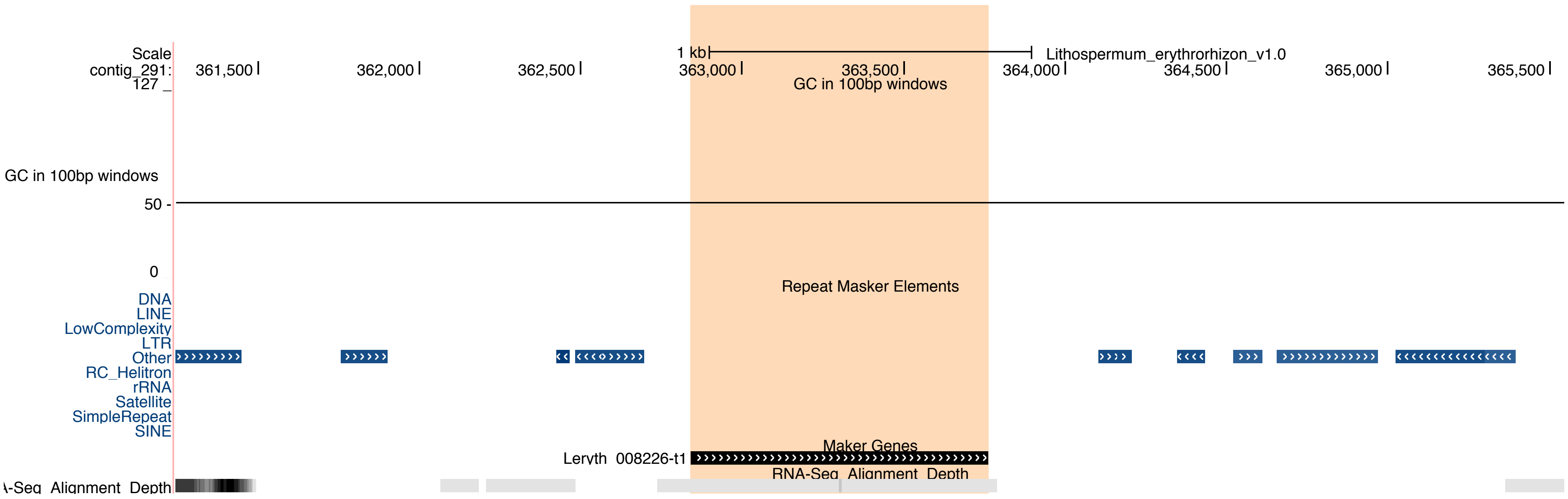

Figure S2c: UCSC Genome Browser region for PGT-like gene (Leryth_008226). Target gene region is highlighted in orange. Repeat elements are shown in blue. RNAseq alignment depth based on 1kb sliding window.

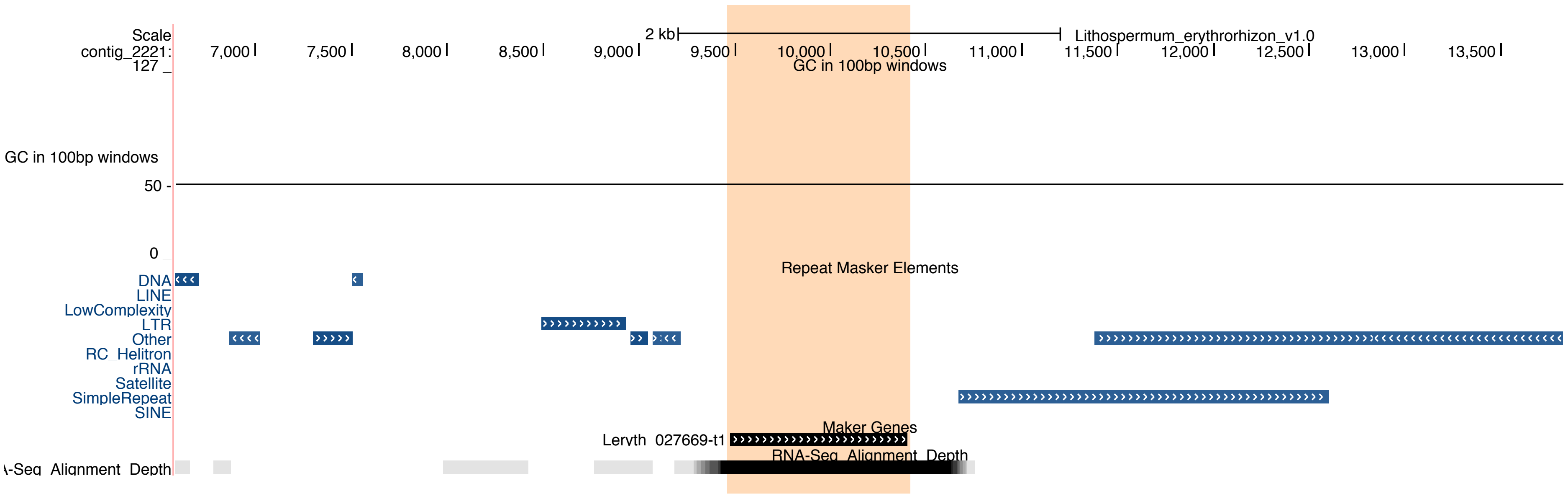

Figure S2d: UCSC Genome Browser region for PGT-like gene (Leryth_027669). Target gene region is highlighted in orange. Repeat elements are shown in blue. RNAseq alignment depth based on 1kb sliding window.

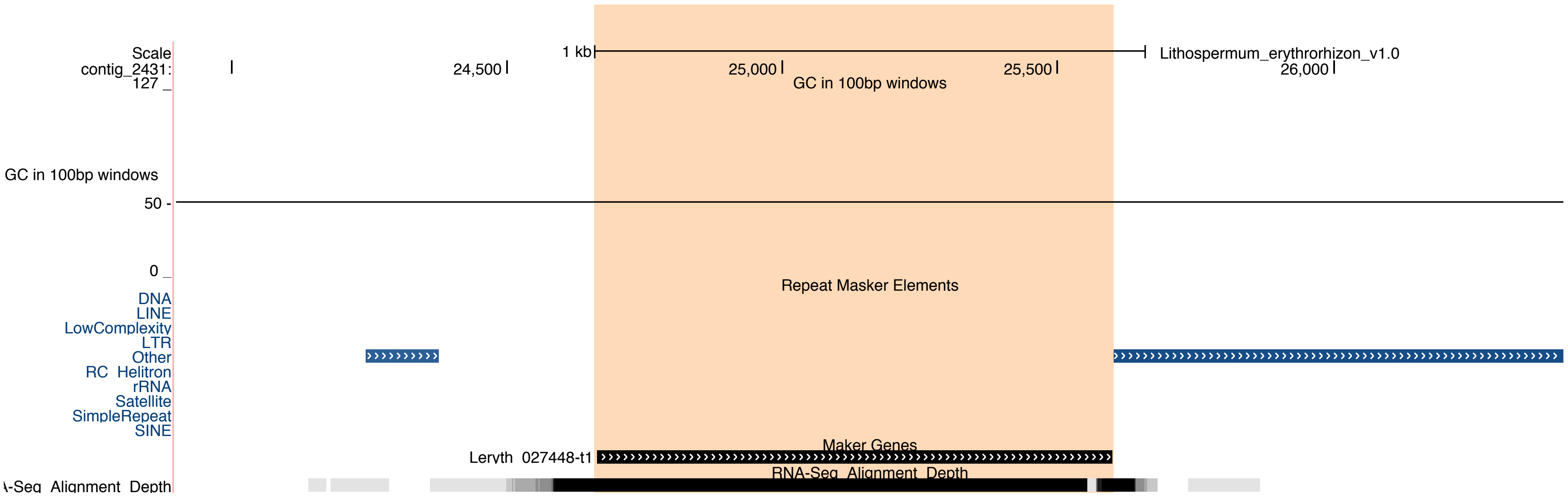

Figure S2e: UCSC Genome Browser region for PGT-like gene (Leryth_027448). Target gene region is highlighted in orange. Repeat elements are shown in blue. RNAseq alignment depth based on 1kb sliding window.

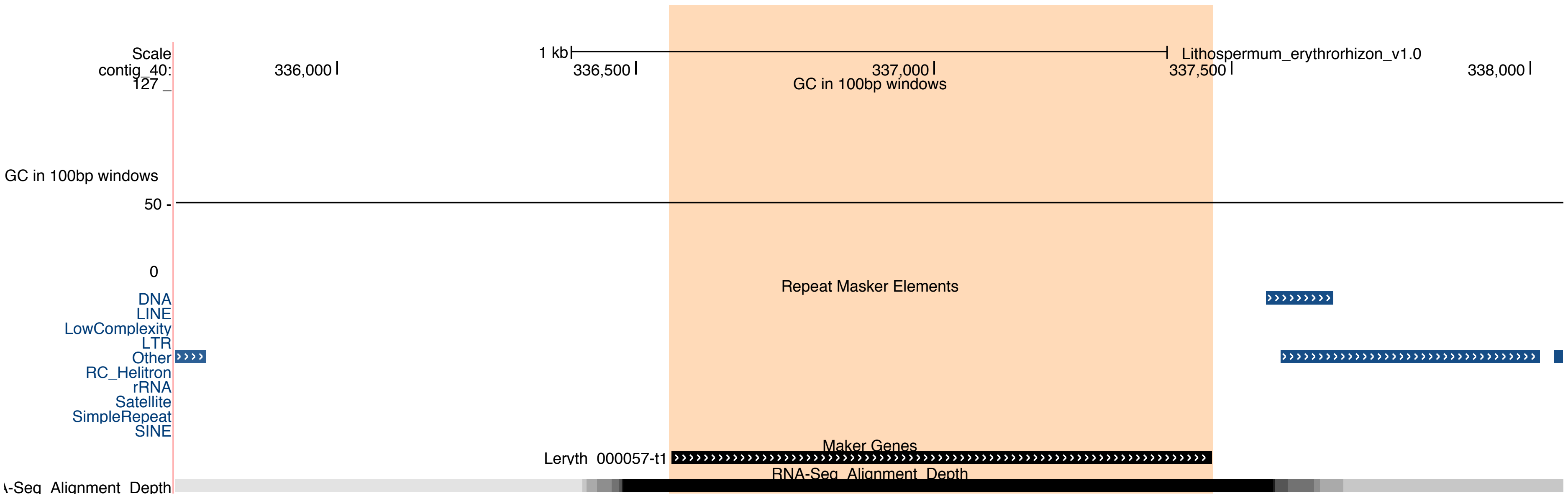

Figure S2f: UCSC Genome Browser region for PGT-like gene (Leryth_000057). Target gene region is highlighted in orange. Repeat elements are shown in blue. RNAseq alignment depth based on 1kb sliding window.

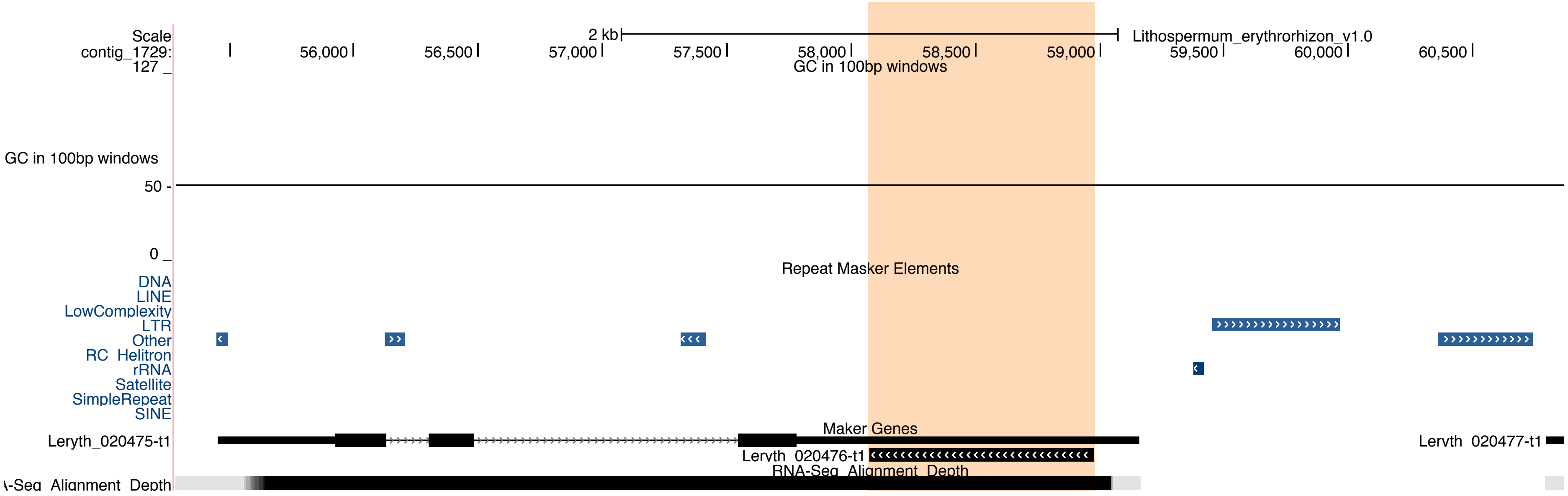

Figure S2g: UCSC Genome Browser region for PGT-like gene (Leryth_020476). Target gene region is highlighted in orange. Repeat elements are shown in blue. RNAseq alignment depth based on 1kb sliding window.

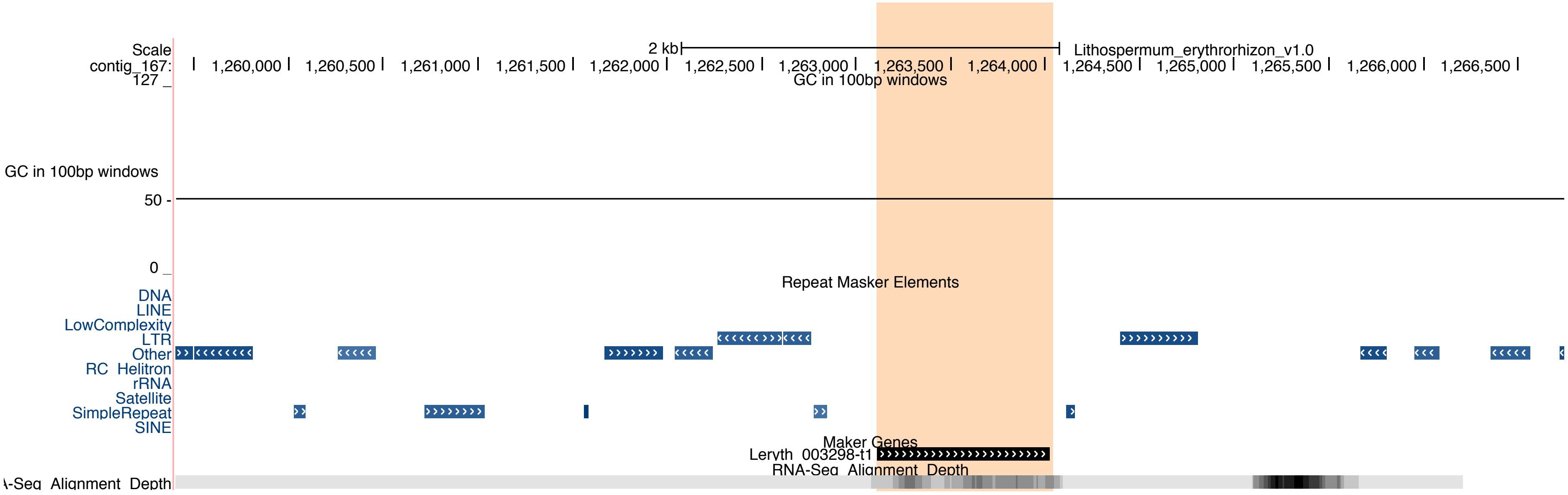

Figure S2h: UCSC Genome Browser region for PGT-like gene (Leryth_003298). Target gene region is highlighted in orange. Repeat elements are shown in blue. RNAseq alignment depth based on 1kb sliding window.

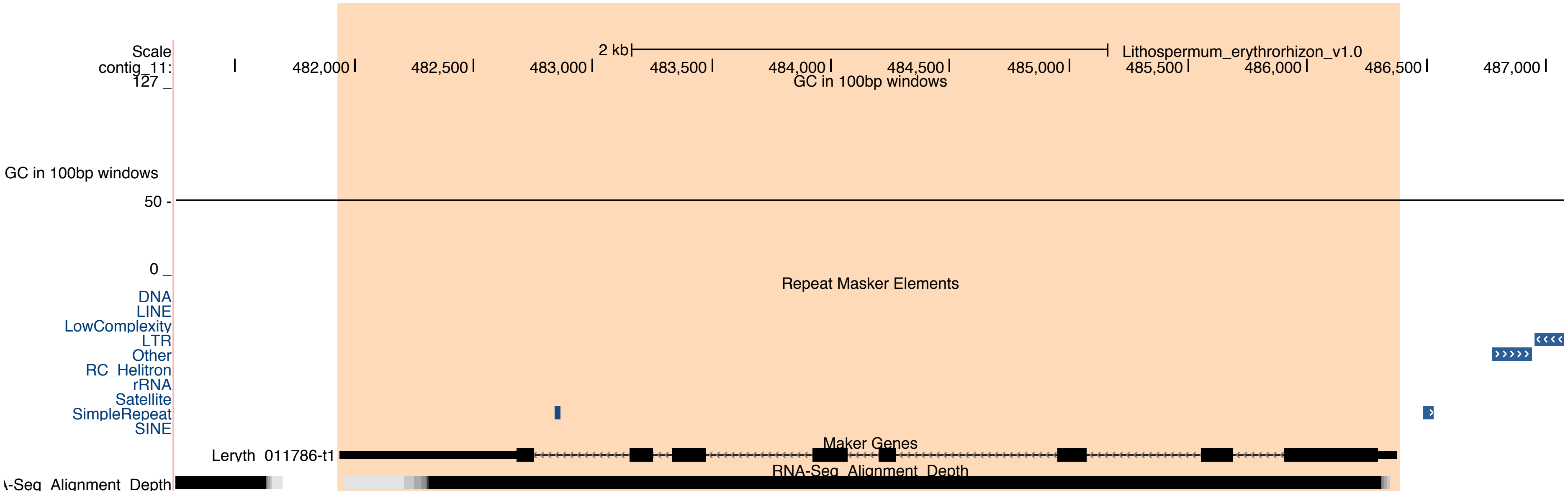

Figure S2i: UCSC Genome Browser region for ubiqinone prenyltransferase-like gene (Leryth_011786). Target gene region is highlighted in orange. Repeat elements are shown in blue. RNAseq alignment depth based on 1kb sliding window.

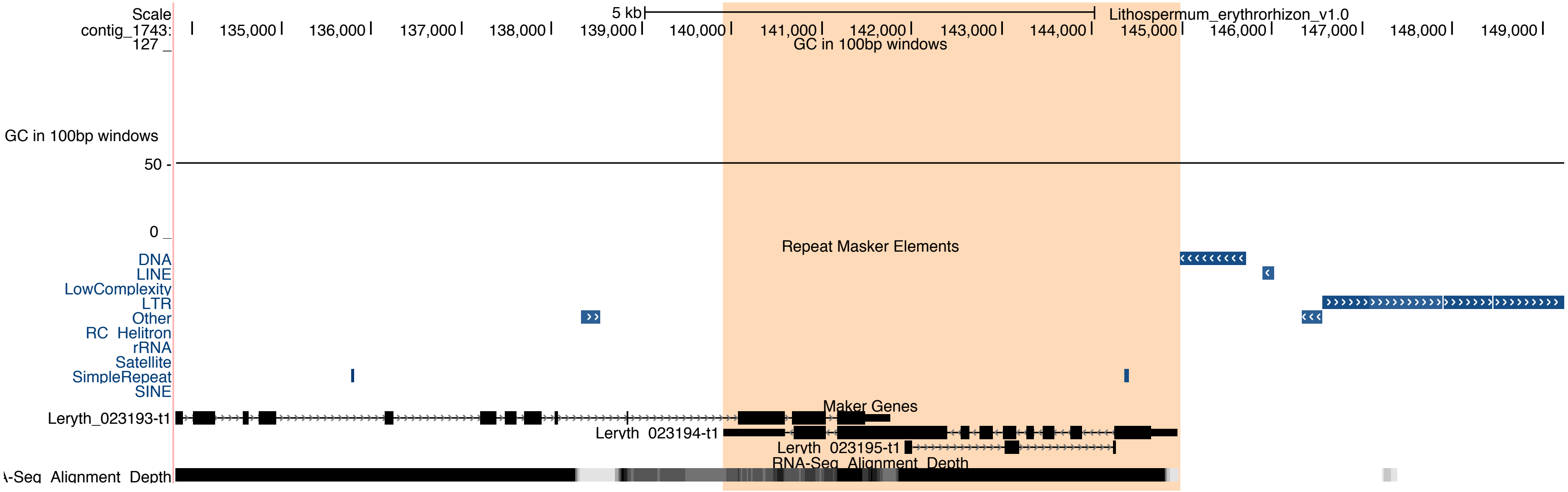

Figure S2j: UCSC Genome Browser region for unclassified prenyltransferase gene (Leryth_023194). Target gene region is highlighted in orange. Repeat elements are shown in blue. RNAseq alignment depth based on 1kb sliding window.

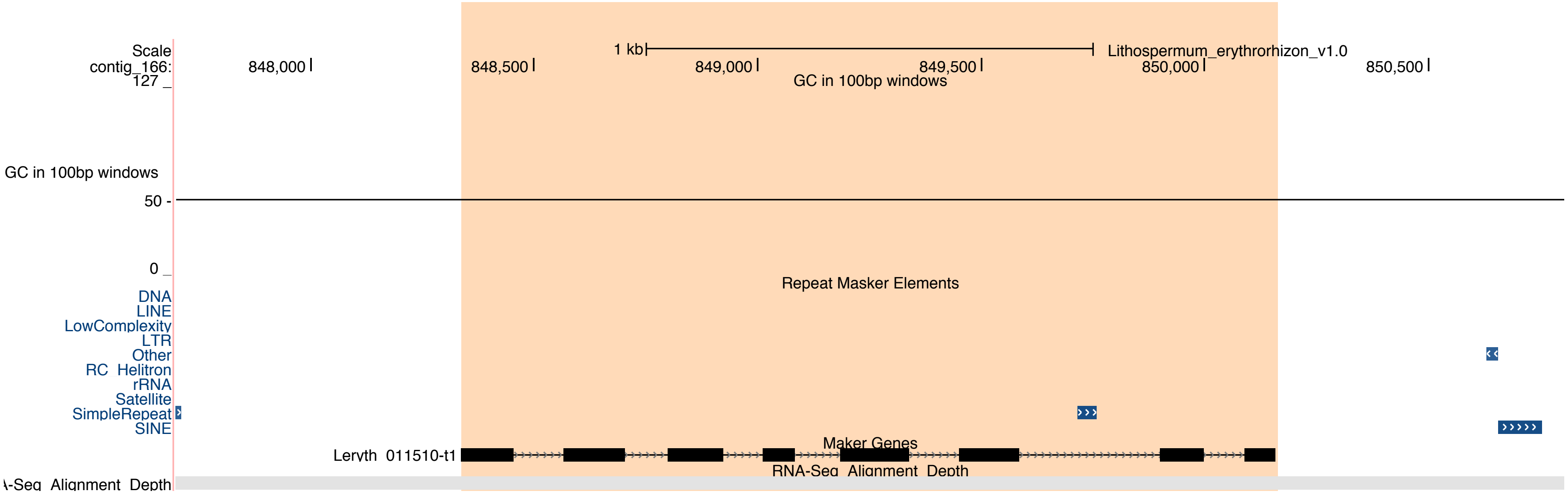

Figure S2k: UCSC Genome Browser region for unclassified prenyltransferase gene (Leryth_011510). Target gene region is highlighted in orange. Repeat elements are shown in blue. RNAseq alignment depth based on 1kb sliding window.

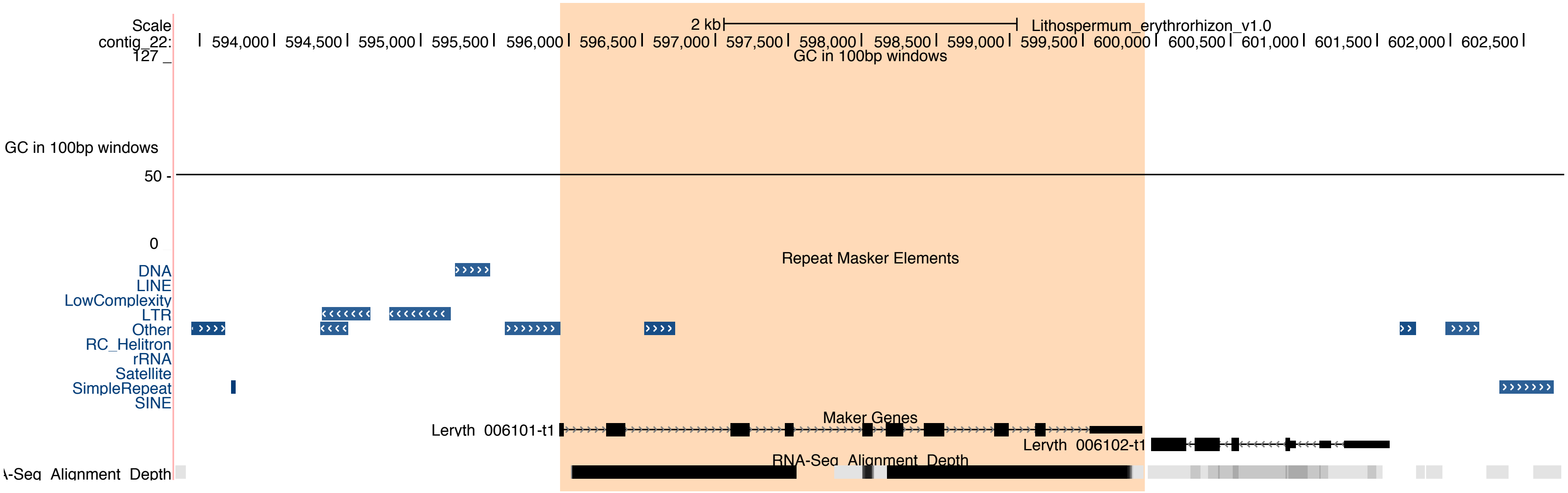

Figure S2l: UCSC Genome Browser region for unclassified prenyltransferase gene (Leryth_006101). Target gene region is highlighted in orange. Repeat elements are shown in blue. RNAseq alignment depth based on 1kb sliding window.

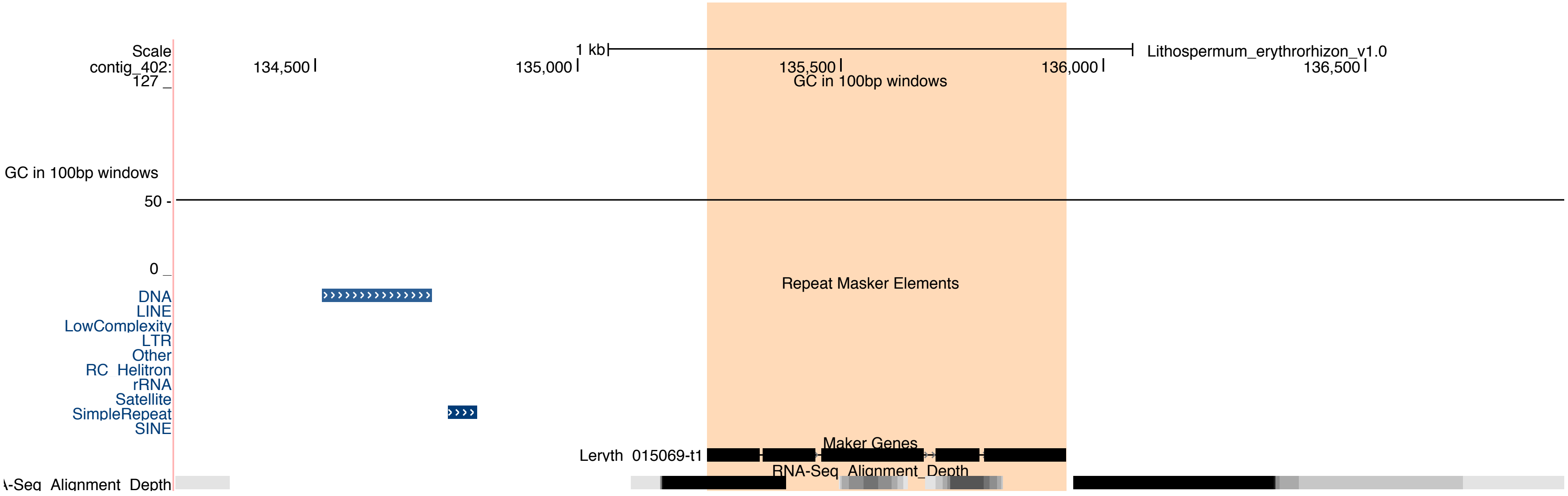

Figure S2m: UCSC Genome Browser region for PGT-like suspected pseudogene (Leryth_015069). Target gene region is highlighted in orange. Repeat elements are shown in blue. RNAseq alignment depth based on 1kb sliding window.
