# Supplementary material for: Hybrid de novo genome assembly of red gromwell (Lithospermum erythrorhizon) reveals evolutionary insight into shikonin biosynthesis
Source: Hortic Res. 2020 Jun 1;7:82. doi: 10.1038/s41438-020-0301-9 (PMC7261806; doi:10.1038/s41438-020-0301-9)
Supplement: Supplementary file 3 — Supplementary Figure 3 [file 41438_2020_301_MOESM3_ESM.pdf]

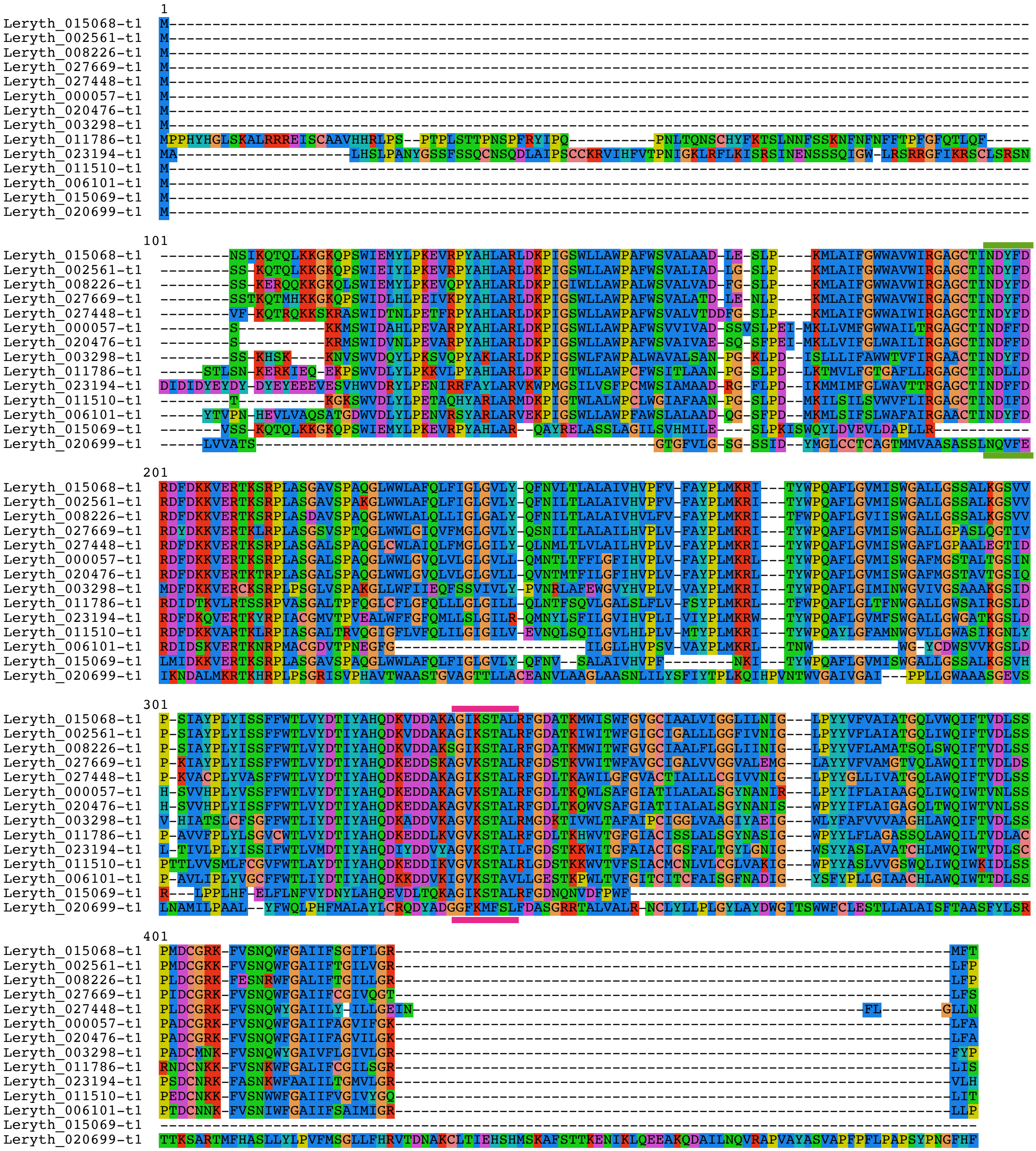

Figure S3. Alignment of all genome assembly proteins with sequence similarity to characterized PGT1 and PGT2. The conserved motif NDXXD indicative of putative prenyl diphosphate binding is indicated by the green bars. The conserved GX(K/Y)STAL motif present in the PGT 4HB:prenyltransferase subfamily is indicated by the pink bars.
