# Supplementary material for: Hybrid de novo genome assembly of red gromwell (Lithospermum erythrorhizon) reveals evolutionary insight into shikonin biosynthesis
Source: Hortic Res. 2020 Jun 1;7:82. doi: 10.1038/s41438-020-0301-9 (PMC7261806; doi:10.1038/s41438-020-0301-9)
Supplement: Supplementary file 4 — Supplementary Figure 4 [file 41438_2020_301_MOESM4_ESM.pdf]

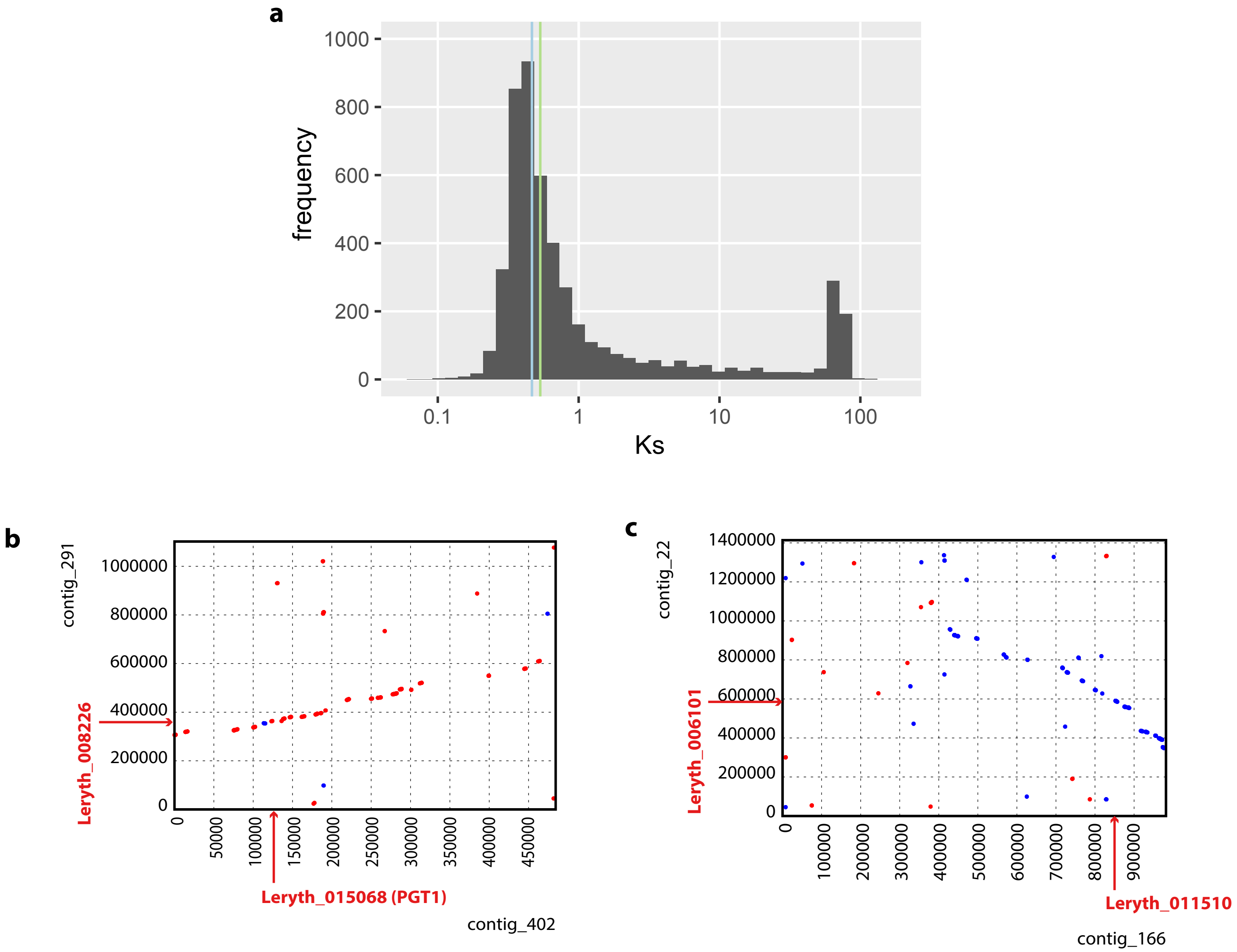

Figure S4. L. erythrorhizon:L.rythrorhizon syntenic block analysis. a) Distribution of synonymous (Ks) substitutions between coding regions with shared synteny. The light green vertical bar indicates the median Ks (0.534) of the syntenic block containing PGT1 (Leryth_01568) and PGT homolog Leryth_008226. Mummerplot of this syntenic block is showin in part b. The light blue vertical bar indicates the median Ks (0.466) of the syntenic block containing PGT homologs Leryth_011510 and Leryth_006101. Mummerplot of this syntenic block is showin in part c. The locations of PGT homologs are indicated by the red arrows.
