# Supplementary material for: Hybrid de novo genome assembly of red gromwell (Lithospermum erythrorhizon) reveals evolutionary insight into shikonin biosynthesis
Source: Hortic Res. 2020 Jun 1;7:82. doi: 10.1038/s41438-020-0301-9 (PMC7261806; doi:10.1038/s41438-020-0301-9)
Supplement: Supplementary file 5 — Supplementary Figure 5 [file 41438_2020_301_MOESM5_ESM.pdf]

a

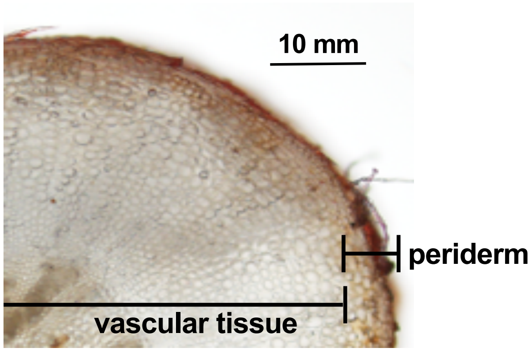

b

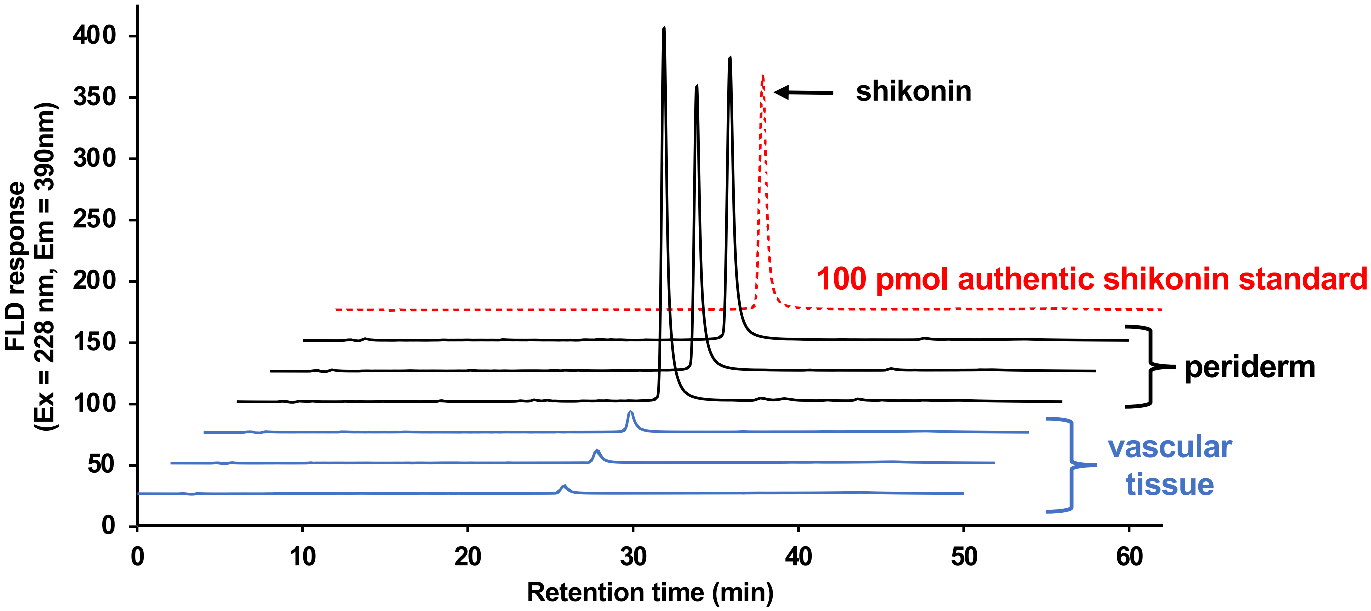

Figure S5. Shikonin is predominantly localized to the periderm of *Lithospermum erythrorhizon* roots. a Cross section of a three-month-old *L. erythrorhizon* root with peeled regions roughly corresponding to vascular tissue and periderm indicated. **b** Chromatograms from HPLC-fluorescence analysis of total shikonins extracted from equal amounts of vascular tissue and periderm. Traces are offset on the x- and y-axes for illustrative purposes. Each trace represents an injection from extractions of three sets of pooled tissues, each prepared from three individuals (n = 3 biological replicates from nine total individuals). The same samples were also used to generate transcriptomes for RNA-seq analysis of periderm and vascular tissues.
