# Supplementary figures and images for: Hybrid de novo genome assembly of red gromwell (Lithospermum erythrorhizon) reveals evolutionary insight into shikonin biosynthesis
Source: Hortic Res. 2020 Jun 1;7:82. doi: 10.1038/s41438-020-0301-9 (PMC7261806; doi:10.1038/s41438-020-0301-9)

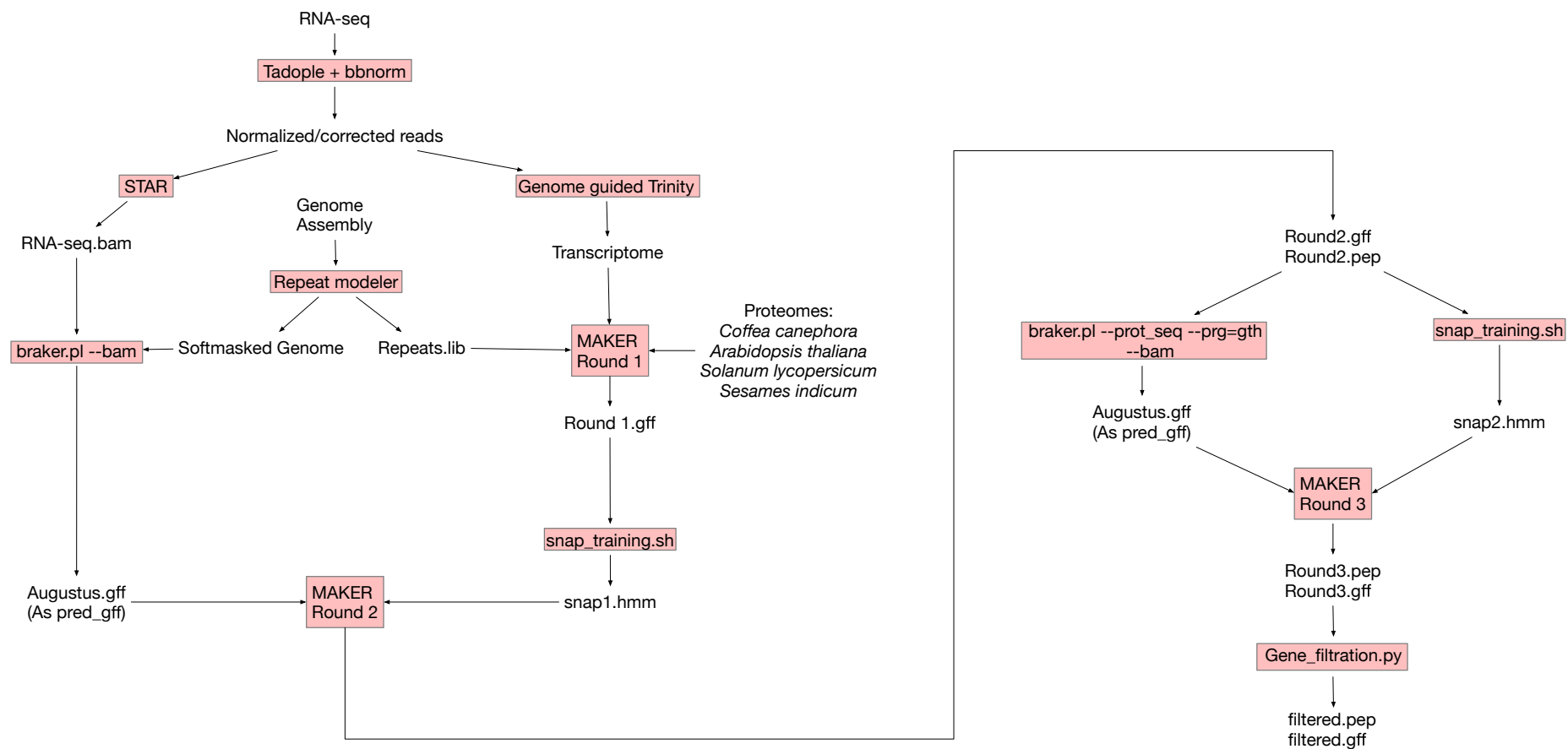

**Figure S6. Flowchart of the *L. erythrorhizon* gene annotation pipeline.**

Supplement: Supplementary file 6 — Supplementary Figure 6 [file 41438_2020_301_MOESM6_ESM.pdf]
